# Floral attractants in the black orchid *Brasiliorchis schunkeana* (Orchidaceae, Maxillariinae): clues for presumed sapromyophily and potential antimicrobial activity

**DOI:** 10.1186/s12870-022-03944-8

**Published:** 2022-12-10

**Authors:** Monika M. Lipińska, Marek Gołębiowski, Dariusz L. Szlachetko, Agnieszka K. Kowalkowska

**Affiliations:** 1 Department of Plant Taxonomy and Nature Conservation, Faculty of Biology, University of Gdańsk, Wita Stwosza 59, 80-308 Gdańsk, Poland; 2 Foundation Polish Orchid Association, 81-825 Sopot, Poland; 3 Department of Environmental Analytics, Faculty of Chemistry, University of Gdańsk, Wita Stwosza 63, 80-308 Gdańsk, Poland; 4 Department of Plant Cytology and Embryology, Faculty of Biology, University of Gdańsk, Wita Stwosza 59, 80-308 Gdańsk, Poland

**Keywords:** 2,5-di-tert-butyl-1,4-benzoquinone, *Maxillaria*, Sapromyophily, Neotropics, Orchids, Pollination

## Abstract

**Background:**

Orchids have evolved various strategies that aim to ensure their reproduction success. These may include the production of rewards for pollinators, or on the contrary, deception. Specific sets of features such as flower morphology, color, nectar, and odor presence (or lack thereof) are considered to determine suitability for pollination by different groups of animals. Stingless bees are thought to be the primary pollinators of the orchids of the Neotropical subtribe Maxillariinae. However, almost black flowered *Brasiliorchis schunkeana* at first glance presents floral adaptations that may suggest another pollination syndrome—sapromyophily.

**Results:**

A few traces of secretion were noticed on the glabrous lip callus and lip apex built by conical to villiform papillae (SEM analysis). Histochemical studies revealed huge amounts of lipids in the epidermis, subepidermis, and some parenchyma cells (SBB test) with various stages of lipids accumulation between cells. Further TEM analysis showed a heterogeneous (lipoid and phenolic) nature of secretion. The dense osmiophilic cytoplasm contained organelles (RER, free ribosomes, dictyosomes, plastids with plastoglobuli, nucleus) and vesicles migrating to plasmalemma. The vesicles, osmiophilic globules, and flocculent material were visible in periplasmic space. The central vacuole possessed osmiophilic phenolic content and flocculent material. GC–MS analysis revealed in floral extract the presence of 7,9-di-tert-butyl-1-oxaspiro(4,5)deca-6,9-diene-2,8-dione (77.06%) and 2,5-di-tert-butyl-1,4-benzoquinone (16.65%). Both compounds are known for their biological activity.

**Conclusions:**

The juxtaposition of results led us to the conclusion that the labellar tissue produces lipoid and phenolic material, which is responsible for the glossiness and rotten herring scent. This type of secretion could be classified as a phenolic resin. The chemical analysis revealed the presence of five semiochemicals that are known to be attractants for some Diptera, which together with the rest of the results constitutes a strong premise that representatives of this order could be potential pollinators of *B. schunkeana*. Field observations however are still needed to confirm this pollination syndrome.

**Supplementary Information:**

The online version contains supplementary material available at 10.1186/s12870-022-03944-8.

## Background

Subtribe Maxillariinae Benth. is one of the richest taxa in the family Orchidaceae Juss. However, its generic circumscription has been under discussion practically since its formal description [1]. For a long time, it has been suspected that it is an assemblage of taxa, consisting of morphologically disparate groups of species [2]. It is said that *Maxillaria *sensu lato covers about 4/5 of the species belonging to the subtribe and counts up to even 750 species [3]. The lack of clearly defined boundaries of *Maxillaria* Ruiz & Pav. resulted in proposing several taxonomic approaches to the subtribe Maxillariinae over the past 150 years.

Stingless bees (Meliponini) are thought to be the main pollinators of the subtribe Maxillariinae [4, 5]. However, visits of other pollinators have been also recorded, *i.e.* bees from the subtribe Euglossini or ants from the subfamily Ponerinae. It is predicted that more than 50% of Maxillariinae representatives attract pollinators with the so-called empty promises—the combination of visual, tactile, and olfactory stimuli [6]. Among the species offering some kind of reward to their pollinators, there are three types of thereof: nectar, pseudopollen (farina), and wax-like substances [7]. Nectar production has been proved in several taxa such as *Ornithidium coccineum* (Jacq.) Salisb. *ex* R.Br. [8], *O. fulgens* [9], and *Maxillaria anceps* Ames & C. Schweinf [6]. Davies et al. [6] estimate that within the core of the subtribe only 8% of species produce nectar.

On the lip surface of some species of the *M. grandiflora*, *M. splendens,* and *M. discolor* alliances, pseudopollen (farina) may be observed. It has a form of whitish mealy coating and is produced by the fragmentation of the labellar trichomes in species that do not offer nectar or any other reward. Some researchers [10, 11] believe that it is collected by bees because of the nutrients it contains such as starch grains, oils, and proteins. According to Davies et al. [6], 16% of the taxa studied by them produce pseudopollen and 7% have trichomes with slightly different construction, but similar functions.

Rewards in the form of wax and resinous substances are produced by floral papillae and trichomes located on the surface of the lip. They are rich in lipids and aromatic amino acids and occur in about 13% of Maxillariinae species [6]. Van der Pijl & Dodson [10] suggested that these substances are collected by bees as a material for nest building. Davies et al. [7] have noticed, however, that due to their nutritional value, waxes and resins can also be a source of food substances.

So far, there are no records of pseudocopulation in the core of the subtribe Maxillariinae. Singer [12] described it as one representative of the segregated taxa—*Trigonidium obtusum* Lindl. Ornithophily, another interesting pollination syndrome, was longly suspected to occur in Maxillariinae. More than 50 years, the conclusive evidence was lacking and all reports on this topic were based on a single observation made by van der Pijl & Dodson [10] who reported the hummingbird *Panterpe insignis* visiting an unidentified species of *Maxillaria *sensu lato with pink, tubular flowers. Finally, in 2022, the ornithophily in the subtribe Maxillariinae has been proven. Crucial observations have been made during the field research in Guatemala, where azure-crowned hummingbirds (*Amazilia cyanocephala*) have been spotted while pollinating flowers of *Ornithidium fulgens* Rchb. f. This finding was later supported by the results of micromorphological, histochemical, and chemical studies [9].

*Brasiliorchis* R. Singer, S. Koehler & Carnevali has been segregated in 2009 from *Maxillaria *sensu lato. It is a small genus that comprises only about 13 to 25 species [13]. Its distribution range covers primarily Brazil, where all species occur within the Atlantic Rain Forest Biome or Mata Atlantica [14, 15], from the Rio Grande do Sul [16] to Bahia [17]. Only three species are known to occur outside Brazil and these are *B. picta* (Hook.) R.B. Singer, S. Koehler & Carnevali, *B. chrysantha* (Barb. Rodr.) R.B. Singer, S. Koehler & Carnevali (both reaching extreme northeastern Argentina), and *B. marginata* (Lindl.) R.B. Singer, S. Koehler & Carnevali (reported from Ecuador; [18] and references therein). Two species: *B. kautskyi* (Pabst) R. Singer, S. Koehler & Carnevali and *B. schunkeana* (Campacci & Kautsky) R. Singer, S. Koehler & Carnevali are endemic to the Brazilian state of Espirito Santo [19]. The genus can be easily distinguished by a set of consistent morphological features. Pseudobulbs are oblong-ovoid, aggregated or distant, sulcate, bifoliate, and subtended by non-foliaceous sheaths. Leaves are linear to elliptic-lanceolate, acute, and leathery. Inflorescences are several and they are produced simultaneously from the base of the most recent pseudobulb. Floral bracts are almost always shorter than the pedicel and ovary. Flowers are campanulate with dissimilar sepals and petals. Lip is always markedly 3-lobed, with usually rounded lobes. The callus is oblong and prominent in the lower half of the lip. Column foot can be either short or long.

*Brasiliorchis schunkeana* was first discovered in 1993. As already mentioned, its distribution range is restricted to the Espirito Santo State, southeastern Brazil, where it grows in the coastal Atlantic rainforest at elevations of 600 to 700 m, as a small-sized, warm-growing epiphyte [19]. *B*. *schunkeana* is characterized by fusiform-cylindric pseudobulbs that are enveloped basally by deciduous leaf sheaths carrying two, apical, erect, linear, conduplicate leaves. This unique species blooms in Brazil in the summer, on a basal, short, single-flowered inflorescence arising on a mature pseudobulb and holding the flower at pseudobulb height. *B. schunkeana* is considered to be the only natural black orchid [20]. Indeed, the coloration of its flower is the closest to the black color, but it is actually a very dark purple-red, which gives the impression of a black flower.

**Fig. 1:**
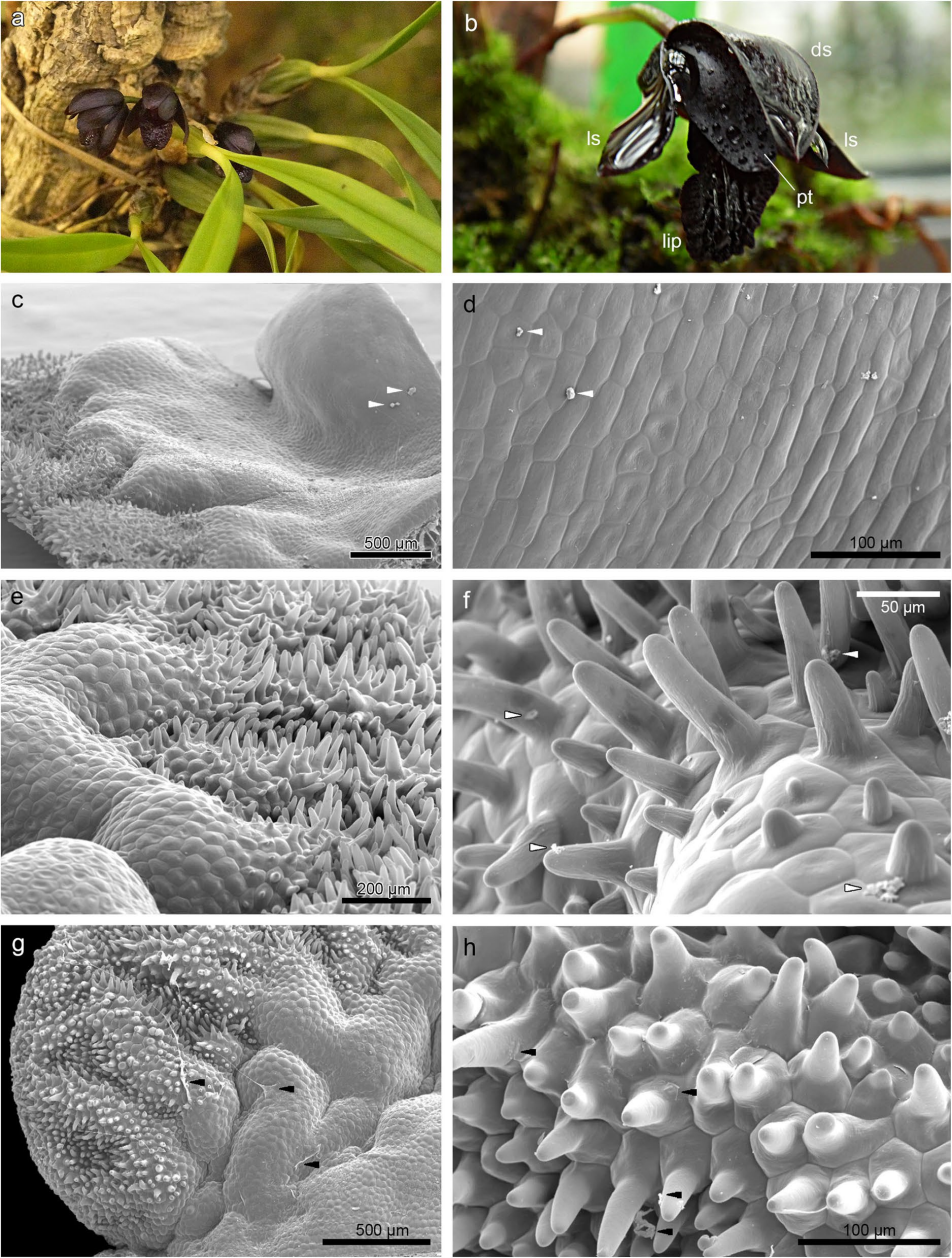
*Brasiliorchis schunkeana*. **a** general habit. **b** single flower: dorsal sepal (*ds*), lateral sepal (*ls*), petal (*pt*), lip; Micromorphological features of the lip (SEM): **c** glabrous lip callus with a few traces of secretion. **d** magnification of **c**, callus surface with secretory remnants. **e** rippled surface and densely papillated distally. **f** conical to villiform papillae with traces of secretion. **g** rippled and papillate lip apex. **h** magnification of the papillae. *Arrows* in **c-d**, **f–h** indicate the traces of secretion

The main aim of the presented study was to comprehensively investigate floral attractants of *Brasiliorchis schunkeana* (Fig. 1a-b) and test the hypothesis that the flowers of this species possess adaptations characteristic for the myophilous or sapromyophilous species, which would constitute the first empirical evidence of possibly fly-pollinated taxon within the megadiverse subtribe Maxillariinae. The secondary goal was to determine if and if so which components of the secretions examined by GC–MS are active compounds of potential medical significance.

## Results

### Micromorphology

The lip was adorned with elevated callus, which begins from the lip base and runs until 1/3 of the lip. The examination in the scanning electron microscope (SEM) revealed a glabrous surface of lip base and callus (Fig. 1c-d). From a small depression underneath the callus, the surface rose upwards and was rippled and densely papillated distally (Fig. 1d-e). The cells of lip margins had conical to villiform papillae (Fig. 1f), conical ones were also present on the outer (abaxial) lip side. A few traces of secretion were noted on the callus (Fig. 1c-d) and the papillae at the lip apex (Fig. 1g-h).

### Histochemistry

**Fig. 2:**
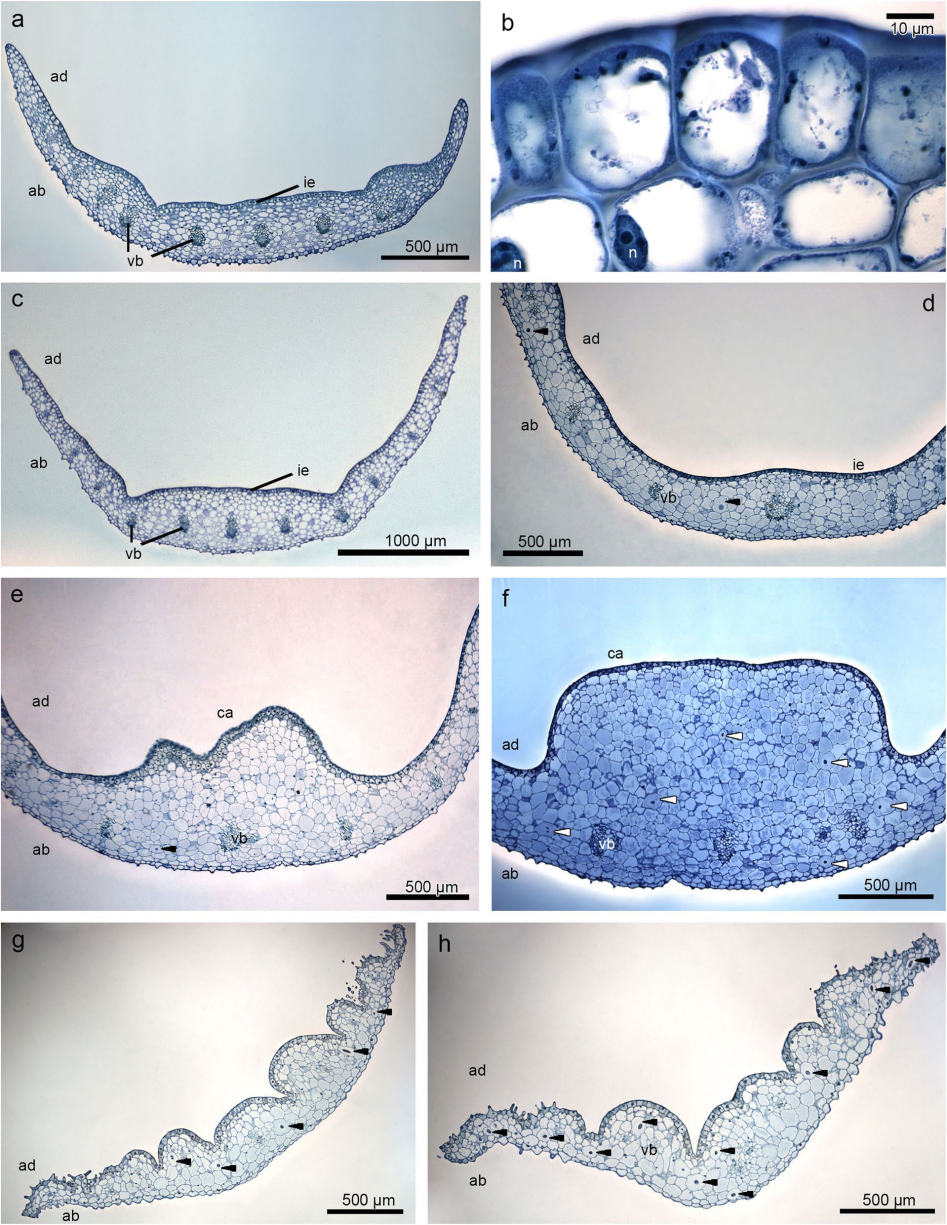
The transverse sections of the lip (TBO, light microscope) show a single layer of the epidermis and few collateral vascular bundles in the parenchyma. **a** lip base with flat callus. **b** epidermis, magnification of **a**. **c** lip—further part. **d-e** lip—beginning of raising callus. **f** rectangular shape of callus. **g-h** lip apex with papillae. *Arrows* indicate idioblasts with raphides. *ab*—abaxial (outer) surface, *ad*—adaxial (inner) surface, *ca*—callus, *ie*—inner epidermis, *n*—nucleus, *pa*—parenchyma, *vb*—vascular bundle

The transverse sections of the flower at the anthesis revealed the single layer of the epidermis, subepidermal layer, and parenchyma with few collateral vascular bundles and idioblasts with raphides that were located at the lip base with flat callus (Fig. 2a-c), through the callus in the middle of the lip (Fig. 2d-f), to the apex (Fig. 2g-h). The callus emerged from the base (Fig. 2d-e) forming a rectangular structure in shape (Fig. 2f). The inner epidermis (adaxial) was glabrous on most of the lip surface. Papillae appeared distally on the labellar margins (Fig. 2g-h). The external (abaxial) epidermis was slightly covered by conical papillae on the whole lip surface (Fig. 2a, c-h).

**Fig. 3:**
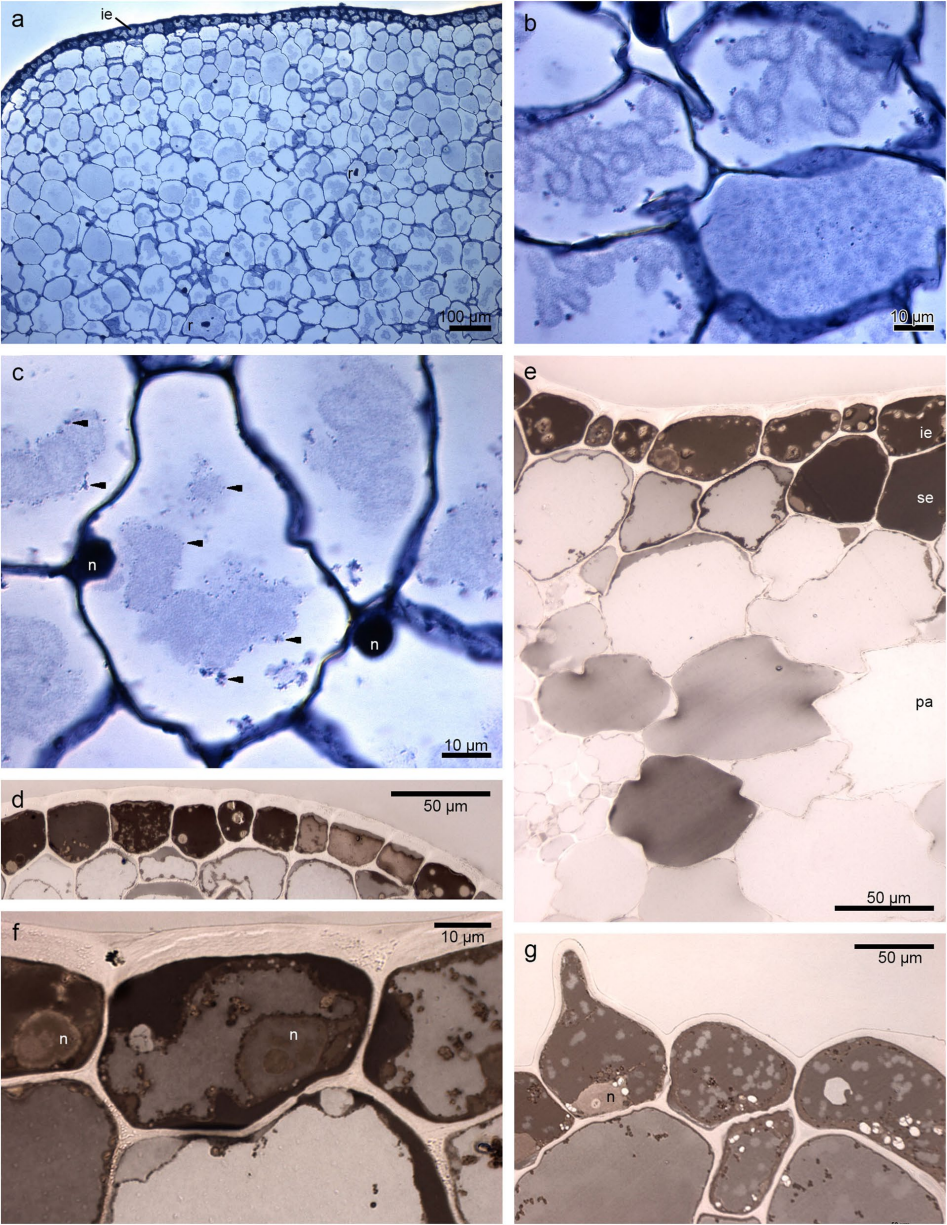
Results of histochemical tests performed on the end of lip callus: **a** single-layered epidermis and idioblasts in parenchyma cells (localized in the middle and at the bottom of tissue, TBO). **b-c** the irregular globules with heterogeneous nature (*arrows*) differing between cells (magnifications of **a**). **d-f** huge amounts of lipids in the epidermis, subepidermis, and in some parenchyma cells (SBB) with the different levels of black color between cells reflecting the various stages of lipids’ accumulation. **g** papillate epidermis of lip apex stained for lipids (SBB). *ie*—inner epidermis, *n*—nucleus, *pa*—parenchyma, *r*—idioblasts with raphides, *se*—subepidermis

The set of histochemical tests gave insight into the localization of different groups of compounds in floral tissue. Besides callus features such as a single-layered epidermis and idioblasts in parenchyma cells (localized in the middle and at the bottom of tissue), our attention was caught by the irregular globules with heterogeneous nature (Fig. 3a-c), visible in varying amounts in cells. The test for lipid detection (SBB) displayed huge amounts of lipids in the epidermis and some parenchyma cells (in callus and lip apex: Fig. 3d-g). Noteworthy was the different levels of black color between cells (Fig. 3f), which can reflect the various stages of lipids’ accumulation. Proteins were slightly stained in the epidermis (Supplementary file 1: Fig. S1a-b) and some cells of the parenchyma (Fig. S1a). Starch grains were few and tiny in size (PAS) (Fig. S1c). The idioblasts with raphides encircled by sheath were present in the whole length of the callus—from base of callus to its end (Fig. S1d-e) and lip apex (Fig. S1f). They were found in the parenchyma from the middle part to the abaxial surface, not in subepidermis. Dihydroxyphenols (FeCl_3_ test) were stained only in plastids (Fig. S1g), possibly in plastoglobules. Ruthenium Red did not detect any mucilage/pectic acids on the surface (Fig. S1h).

### Ultrastructure

**Fig. 4:**
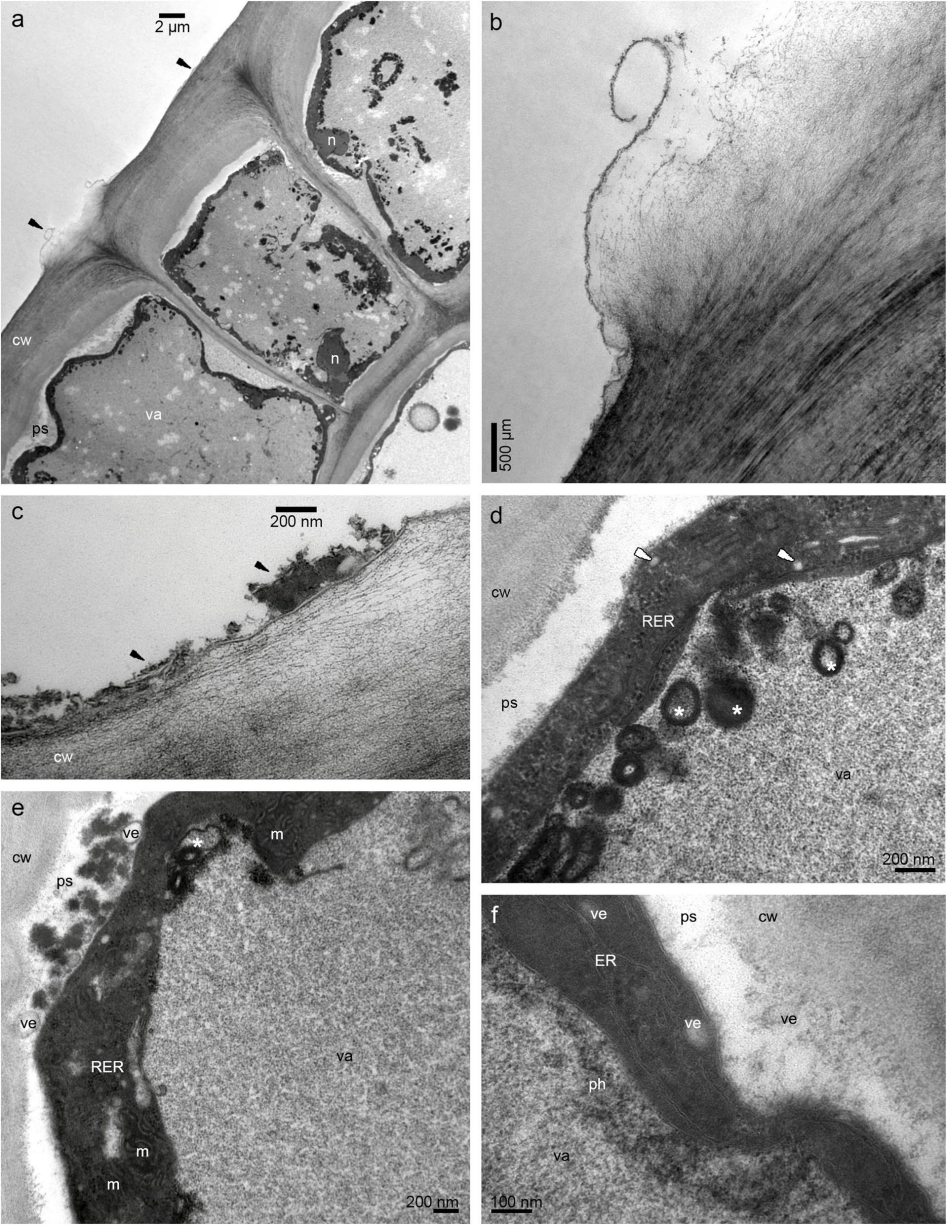
The observations of the epidermis of lip base with flat callus from transmission electron microscopy (TEM) showing: **a** a few residues of secreted materials on the cuticle surface, especially between radial cell walls, thick outer tangential cell walls. **b** magnification of **a**, ruptured cuticle caused by the accumulation of secretion beneath. **c** heterogeneous (lipoid and phenolic) nature of secretion. **d** periplasmic space occurred, dense osmiophilic cytoplasm with organelles and vesicles (*white arrows*), the central vacuole with vesicles with osmiophilic content (*asterisks*). **e** periplasmic spaces with few vesicles, flocculent material, and globules with osmiophilic material. **f** vesicles in periplasmic space and in electron-dense cytoplasm, and osmiophilic, phenolic content in the vacuole. *cw*—cell wall, *m*—mitochondrion, *n*—nucleus, *ph*—phenolic content, *ps*—periplasmic space, *RER*—rough endoplasmic reticulum, *va*—vacuole, *ve*—vesicle

**Fig. 5:**
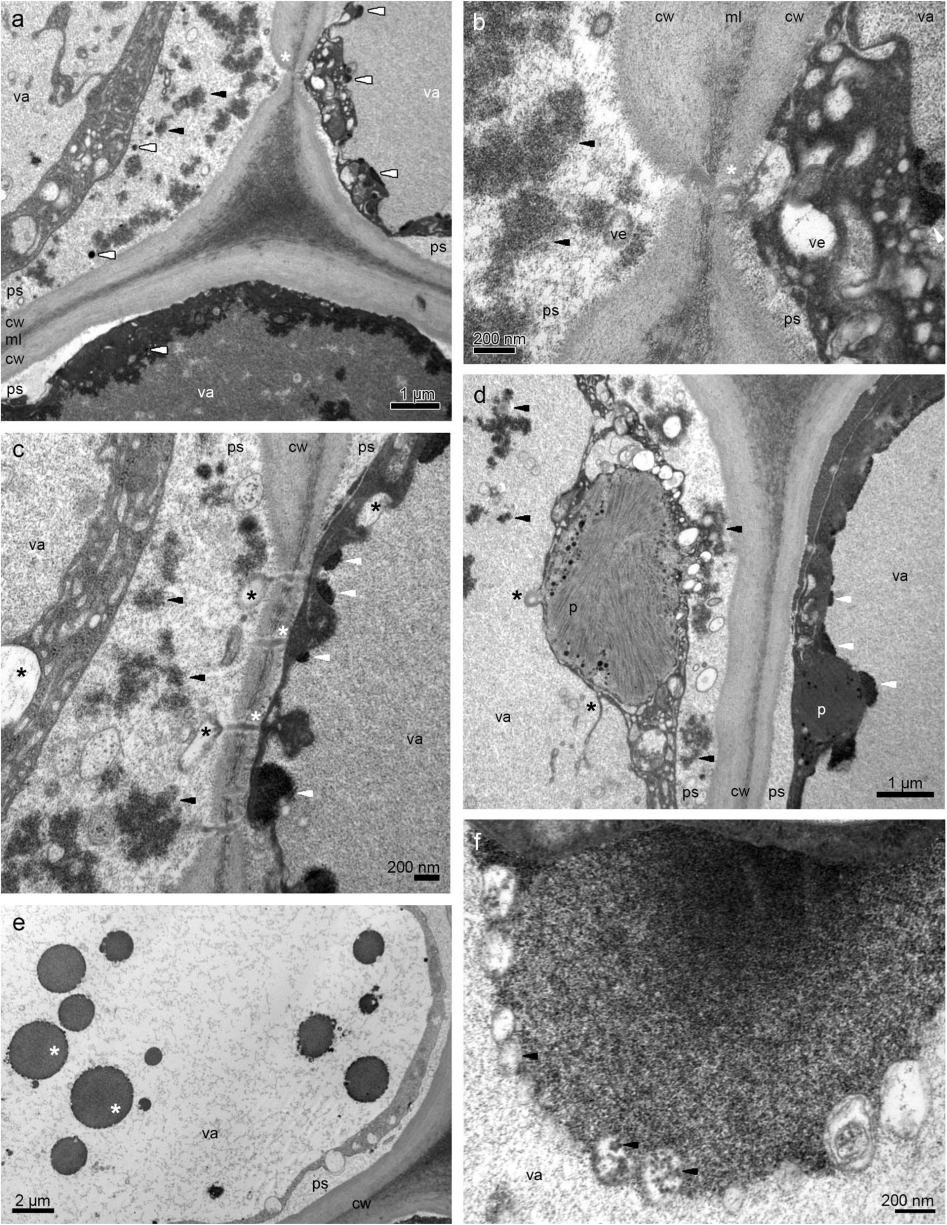
The further observations of the epidermis of lip base with flat callus from transmission electron microscopy (TEM) show: **a** plasmodesmata (*asterisk*) joining the protoplasts of cells, flocculent osmiophilic lipoid/phenolic material (*black arrows*), osmiophilic lipoid globules in periplasmic space and osmiophilic phenolic content in the vacuole (*white arrows*). **b** magnification of **a**. **c** plasmodesmata between cells, osmiophilic phenolic content in the vacuole (*white arrows*), vesicles in the cytoplasm and periplasmic space (*asterisks*), flocculent osmiophilic phenolic material, sometimes formed in globules (*black arrows*). **d** plastid with lamellae and plastoglobules, vesicles close to the plastid (*asterisks*), flocculent osmiophilic phenolic material (*black arrows*) in the vacuole and periplasmic space, osmiophilic phenolic content in the vacuole (black arrows). **e** subepidermal cell with osmiophilic annular profiles (fully filled with the material) (*asterisks*) and flocculent precipitates in the vacuole. **f** osmiophilic content in the vacuole, with numerous vesicles transferring such material inside (*arrows*). *cw*—cell wall, *ml*—middle lamella, *p*—plastid, *ps*—periplasmic space, *va*—vacuole, *ve*—vesicle

The examination of samples from the lip base with flat callus (compare with Fig. 2c) in transmission electron microscopy (TEM) revealed a few residues of secreted materials on the cuticle surface, especially between radial cell walls (Fig. 4a-d). The outer tangential walls were thick. The cuticle was sometimes ruptured (Fig. 4b), which was probably caused by the accumulation of secretion beneath. The secretory products were heterogeneous (Fig. 4b-c): lipoid and phenolic (compare with Tab. 1). The protoplast was dense, fulfilled with lipids, and the central vacuole had phenolic content (Fig. 4d). The cuticle was thin, with no micro-channels (Fig. 4a-d). In cuboidal or rectangular epidermal cells, periplasmic spaces (Fig. 4a) had few vesicles and flocculent material (Fig. 4d-f), also globules with osmiophilic material (Fig. 4e). The vesicles were visible in periplasmic space and in dense, osmiophilic cytoplasm (Fig. 4f). The cytoplasm (Fig. 4a, d-f) was filled with organelles: plastids with plastoglobules and profiles of the smooth and rough endoplasmic reticulum. The large vesicles contained osmiophilic borders in the vacuole (Fig. 4d-e). The material in cytoplasm and vacuoles in different epidermal cells varied greatly, which was caused by different levels of lipids and phenols accumulation, respectively (Fig. 4a, S2a, S2c, compare with Fig. 3d-g). Also, depending on the stage of secretion the periplasmic spaces appeared narrow or expanded, with varying quantities and sizes of globules and vesicles as well as other materials passing through it to the exterior (Fig. 4d-f and S2a-d). The large vacuolar globules, sometimes disintegrated, were illustrated in Fig. S2c, e. In the thin parietal layer of dense cytoplasm the organelles were slightly visible: plastids—chromoplasts (containing lamellae, osmiophilic plastoglobules, Fig. 5d, and starch grain, Fig. S2e-f), the numerous profiles of the rough endoplasmic reticulum, the mitochondria (Fig. S2f). Near them in one cell osmiophilic (lipoid and/or phenolic) material was gathered in periplasmic space, and in the second one—cytoplasmic lipoid and vacuolar phenolic materials (Fig. 5a-c). The starch grains were sometimes utilized in chromoplasts (Fig. 5d, compared with S2e). In subepidermal cells, the vacuoles also contained osmiophilic annular profiles (however, completely filled with the material) and flocculent phenolic precipitates in the vacuole (Fig. 5e). The osmiophilic compounds could be transported through vesicles, as their content was noticeable inside of them (Fig. 5f).

**Table 1:** The main results of micromorphological, histochemical and ultrastructural features of flowers of *Brasiliorchis schunkeana*, and chemical composition of lips

Floral part and surface/method	Micromorphology (SEM)	Histochemistry (TBO, ABB, SBB, PAS, FeCl_3_, Ruthenium Red)	Ultrastructure (TEM)	Chemical composition (GC/MS)
*adaxial (inner) surface*				
**lip base**	glabrous surface	huge amounts of lipids (SBB), small amounts of proteins (ABB), few, tiny strach grains (PAS), dihydroxyphenols (FeCl_3_)	a few residues of secreted materials, cuticle sometimes ruptured, lipoid and phenolic nature of secretion, lipids in cytoplasm,phenols in vacuoles, periplasmic spaces with vesicles and flocculent material, globules with osmiophilic material, vacuole with large vesicles with osmiophilic borders,	16 compounds in methanolic extract (*i.a.* including fatty acid methyl esters, monoacylglycerols, dicarboxylic acids, glycerol, trehalose), 6 compounds in dichloromethane extract (*i.a.* phenols)
**lip callus**	glabrous surface, a few traces of secretion	idioblasts with raphides (TBO, PAS)	remnants of secretion, phenolic accumulation in vacuoles,	
**lip apex**	conical to villiform papillae, a few traces of secretion	idioblasts with raphides (TBO, PAS)	papillae with osmiophilic phenolic content, vacuoles with osmiophilic phenolic globules, and phenolic content, vacuolar fragmentation	
**lip margins**	conical to villiform papillae			
*abaxial (outer) surface*				
**lip**	conical papillae			
**dorsal sepal**		adaxial (inner) surface with papillae and few sessile trichomes (TBO, PAS), idioblasts with raphides (TBO, PAS), lipids (SBB), dihydroxyphenols (FeCl_3_)		
**lateral sepals**		small amounts of lipids (SBB), few tiny starch grains (PAS),		
**petals**		lipids (SBB)		
**column foot**		dihydroxyphenols (FeCl_3_)

**Fig. 6:**
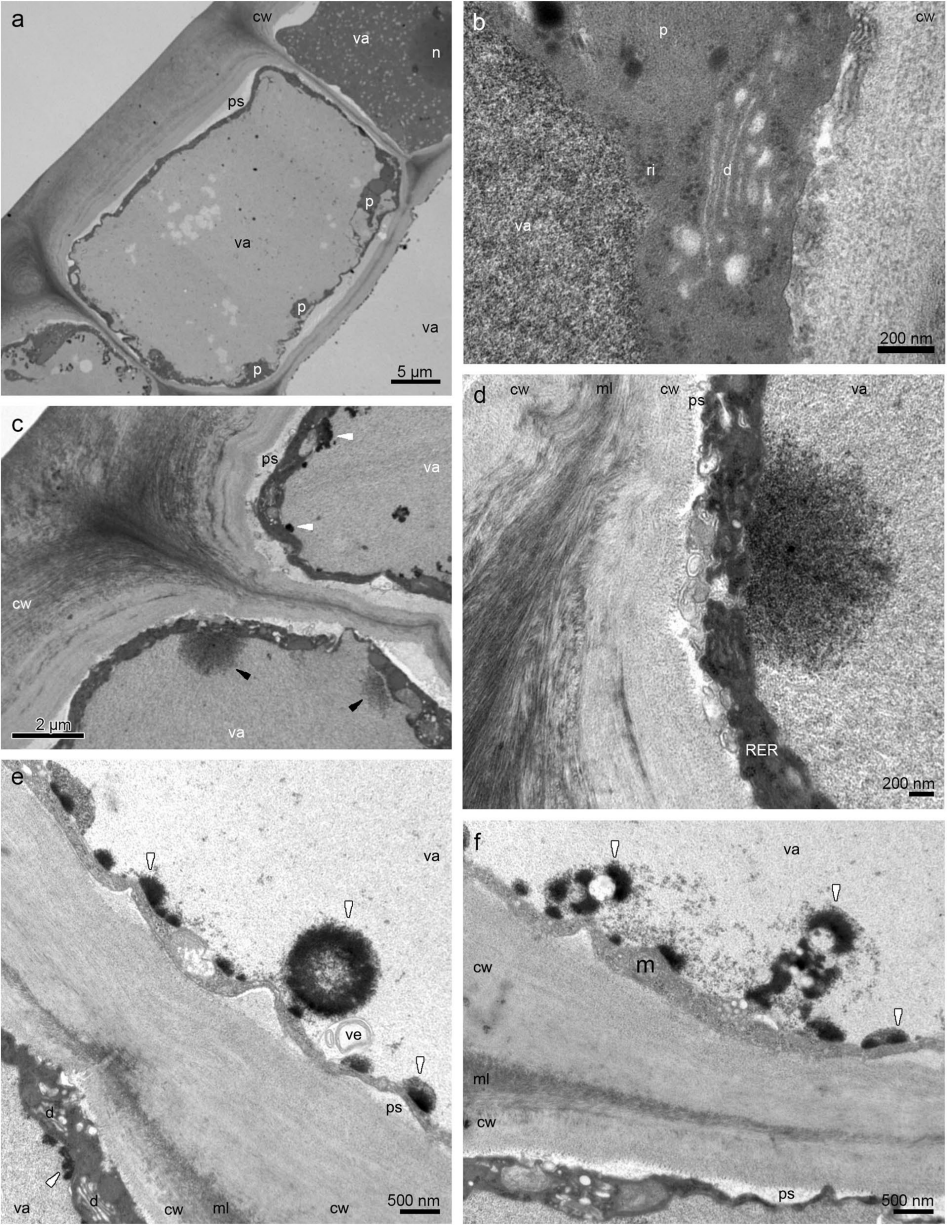
The TEM micrographs of the epidermis of the end of the callus (1/3 of the lip length) from transmission electron microscopy (TEM): **a** cuboidal and rectangular cells with thick, outer tangential walls, different concentrations of compounds in vacuoles, and their fragmentation. **b** magnification of **a**, in the cytoplasm: plastid with plastoglobules, dictyosome, ribosomes, numerous vesicles building into plasmalemma. **c** different levels of osmiophilic phenolic concentration in vacuoles (*white and black arrows*). **d** magnification of **c**. **e–f** osmiophilic phenolic content, sometimes forming globules (*white arrows*) in the vacuole. *cw*—cell wall, *d*—dictyosome, *m*—mitochondrion, *ml*—middle lamella, *n*—nucleus, *p*—plastid, *ps*—periplasmic space, *RER*—rough endoplasmic reticulum, *va*—vacuole, *ve*—vesicle

The callus epidermis at 1/3 of the lip was also built by cuboidal and rectangular cells with thick, outer tangential walls and different concentrations of compounds in vacuoles (Fig. 6a). The remnants of secretion were sporadically present on the surface. Dense, thin, parietal cytoplasm contained chromoplasts with plastoglobules, dictyosomes, rough endoplasmic reticulum, and numerous vesicles building into plasmalemma (Fig. 6b). In Fig. 6c, d, e, f S3a the different levels of osmiophilic phenolic accumulation in vacuoles are illustrated, sometimes formed as globules (Fig. 6e). The large nuclei were noticed in the epidermis, surrounded by numerous plastids and vesicles (Fig. S3b). At the lip apex on the papillae, the heterogenous remnants of secretion with osmiophilic phenolic content (also gathered in vacuole) were shown (Fig. S3c-d). The vacuolar fragmentation, osmiophilic phenolic globules, and phenolic content were visible in vacuoles (Fig. S3e-f).

**Fig. 7:**
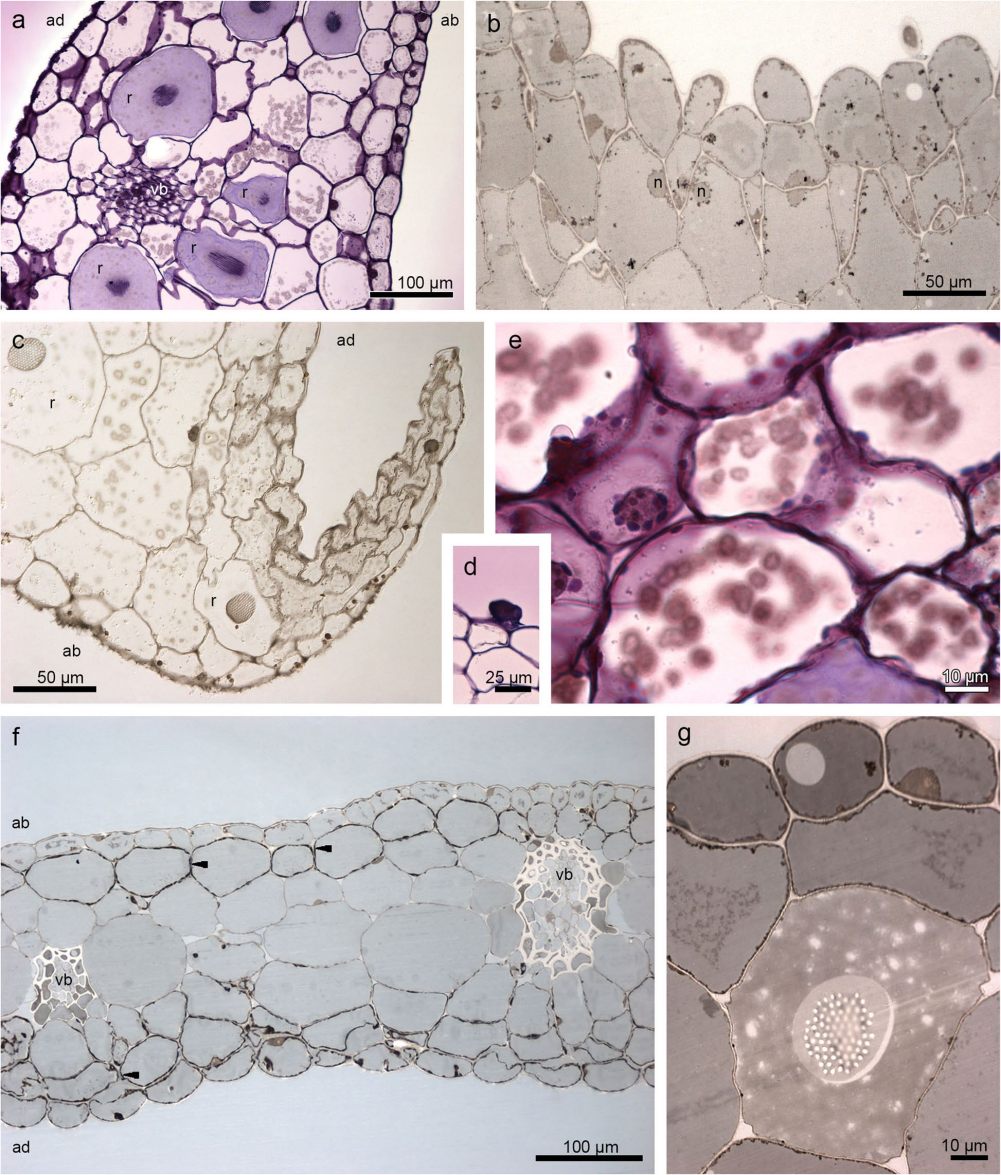
Histochemical tests showing: the transverse sections through dorsal sepal **a** with numerous idioblasts with raphides, especially at the apex. **b** papillate, stained positively on lipids, with lipid droplets (SBB). **c** test with FeCl_3_. **d** sessile trichome present in the middle part of the dorsal sepal. The transverse sections through lateral sepal: **e** PAS. **f** thin parietal cytoplasm stained in SBB. Petal: **g** lipids indicated strongly, similarly as in dorsal sepal and lip (SBB). *ab*—abaxial (outer) surface, *ad*—adaxial (inner) surface, *n*—nucleus, *r*—idioblasts with raphides, *vb*—vascular bundle

The transverse sections through the dorsal sepal revealed numerous idioblasts with raphides, especially at the apex (Fig. 7a). Below the adaxial (inner) surface was built by papillae and stained positively for lipids (Fig. 7b) and dihydroxyphenols (Fig. 7c), and negatively for proteins. In the middle part on the smooth epidermis few sessile trichomes occurred (Fig. 7d). The structure of lateral sepals was the same as in the dorsal sepal (Fig. 7e). The test for the presence of lipids showed fewer lipids in lateral sepals—thin parietal cytoplasm was stained in SBB (Fig. 7f), whereas in petals lipids were indicated more strongly, similarly as in dorsal sepal and lip (Fig. 7g). In sepals, a few tiny starch grains occurred, which is connected with their utilization. The single-layered epidermis of the column foot (Fig. S4a) was stained more intensively for dihydroxyphenols, but not for proteins and polysaccharides (Fig. S4b-d).

The main micromorphological, histochemical and ultrastructural features of flowers of *B. schunkeana* are gathered in Table 1.

### GC–MS analysis

Table 2 summarizes the results obtained during the identification of organic compounds in the methanolic and dichloromethane extracts from lips. A total of 16 compounds were identified in methanolic extract, including fatty acid methyl esters, monoacylglycerols, dicarboxylic acids, glycerol, trehalose, and several other organics. The most abundant compounds in methanolic extract were monoacylglycerols: 1-monostearin (33.50%), 1-monopalmitoylglycerol (30.70%), 2-monostearin (11.22%), and 2-monopalmitoylglycerol (10.14%). Further four were identified in smaller quantities (from 1.0 to 5.0%): glycine, *N*-acetyl (2.79%), glycerol (2.77%), 9,12-octadecadienoic acid, methyl ester (1.83%) and D-( +)-trehalose (3.85%). The remaining compounds were present in quantities below 1.0%.

**Table 2:** Results of the GC–MS analysis. RT stands for retention times

**Methanolic extract**			
No	RT	%	Compound
1*	12.822	2.79	Glycine, N-acetyl-
2	16.371	0.15	Benzoic acid
3	17.625	2.77	Glycerol
4*	20.682	0.66	Pyroglutamic acid
5	25.581	0.09	Hydroxybenzoic acid
6	26.991	0.09	Suberic acid
7	28.851	0.45	Azelaic acid
8	33.847	1.83	9,12-Octadecadienoic acid, methyl ester
9	33.954	0.57	9,12,15-Octadecatrienoic acid, methyl ester
10	34.345	0.24	Octadecanoic acid, methyl ester
11	38.546	0.75	Tetradecanoic acid, propyl ester
12	40.720	10.34	2-Monopalmitoylglycerol
13	41.160	30.70	1-Monopalmitoylglycerol
14	43.171	11.22	2-Monostearin
15	43.599	33.50	1-monostearin
16*	43.777	3.85	D-( +)-Trehalose
**Dichloromethane extract**			
No	RT	%	Compound
1	4.980	0.41	Ethylbenzene
2	5.193	2.57	p-Xylene
3	5.826	1.21	o-Xylene
4*	24.204	2.10	Phenol, 3,5-di-tert-butyl-
5*	33.488	77.06	7,9-Di-tert-butyl-1-oxaspiro(4,5)deca-6,9-diene-2,8-dione
6*	37.055	16.65	2,5-di-tert-Butyl-1,4-benzoquinone

*- tentative identification

Dichloromethane extract contained only six organic compounds. The major components were 7,9-di-tert-butyl-1-oxaspiro(4,5)deca-6,9-diene-2,8-dione (77.06%) and 2,5-di-tert-butyl-1,4-benzoquinone (16.65%). The compounds occurring in smaller quantities (from 1.0 to 3.0%) were: phenol, 3,5-di-tert-butyl- (2.10%), p-xylene (2.57%) and o-xylene (1.21%). Ethylbenzene one was present in a concentration below 1%.

## Discussion

Representatives of the genus *Brasiliorchis* for many years have been considered deceptive and/or rewardless orchids [13, 19]. However, contrary to what has been assumed, research conducted by Pansarin et al. [21] revealed that *Brasiliorchis picta* offers food‐hairs as a reward. Generally, there are only a handful of species within this genus that have been investigated so far, *e.g.* by Flach et al. [22] or Davies & Stpiczyńska [23].

According to the results presented by Davies & Stpiczyńska [23], the callus in all three species of *Brasiliorchis* that they have examined, including *B. schunkeana*, is largely glabrous, and only small, nonsecretory papillae were observed on its surface. In these species, the epidermal cells of the callus were cuboidal in section. As the authors have reported, the labellar callus of *B. schunkeana* had short conical papillae, but the distal, median, and rugose region was glabrous. In our research, however, we have observed that the callus was smooth and the epidermal cells (from the lip base with a flat callus to the 1/3 of the lip where the callus terminates) were cuboidal or rectangular and possessed thick outer tangential walls. Contrary to Davies & Stpiczyńska [23], we have observed conical to villiform papillae at the lip apex, and moreover, conical ones were also present on the outer (abaxial) lip side. As reported in previously published papers, the adaxial labellar surface of *B. picta* was built by conical, subclavate, or villiform papillae whereas in *B. porphyrostele* (Rchb. f.) R.B. Singer, S. Koehler & Carnevali*,* papillae were conical, obconical, or obpyriform. In both species, the glabrous epidermal cells of the callus lacked thick, outer tangential walls [23]. On the cellular level, *B. schunkeana* presents certain similarities with some other representatives of Maxillarinae. For instance, lips of *Maxillariella sanguinea* (Rolfe) M.A. Blanco & Carnevali, *M. variabilis* (Bateman *ex* Lindl.) M.A. Blanco & Carnevali, and *M. vulcanica* (F. Lehm. & Kraenzl.) M.A. Blanco & Carnevali are also built by glabrous callus and papillate labellar margins and apex (conical, obpyriform, villiform papillae) and possess thick tangential walls [24].

On the callus and papillate lip apex of *B. schunkeana* traces of secretion were present in scant quantities (SEM and TEM results). In both *B. picta* and *B. porphyrostele*, the same methods revealed small amounts of secretion on the epidermis, which might be constituents of fragrance [23]. In other representatives of Maxillarinae similar results were obtained, albeit naturally, taxa in this species-rich subtribe show great diversity. Whitten et al. [2] stated that representatives of the genus *Maxillariella, e.g. M. sanguinea* and *M. variabilis* produce only fruity fragrances. Later, Davies & Stpiczyńska [23] revealed that the lips of *M. elatior* and *M. variabilis* secreted resin. Lipińska et al. [24] also observed residues of secreted material in three investigated representatives of *Maxillariella*, however, in different labellar locations and quantities, *e.g.* in *M. sanguinea*, secretory material occurred in high quantities on conical and villiform papillae, but in the scant amount on the callus, whereas in *M. vulcanica* it was accumulated in a depression of callus. The meager quantities of secreted materials on the epidermis are generally explained by the nature of exudated substances. The scent compounds are exuded periodically, vaporize, and do not accumulate on the surface, because of their cytotoxic nature [25], leaving only some residues. In *B. schunkeana*, the thin cuticle was easily cracked by the substances gathered beneath it, especially in the joining cells. However, no micro-channels were observed, likewise in the previously studied species *e.g.* in *M. sanguinea* and *M. variabilis* [24]. Although the micro-channels, which can facilitate the transport across the cuticle to the exterior, were noticed in for instance *M. vulcanica* [24] and in several other orchid species [26–28].

Davies & Stpiczyńska [23] noted the absence of a well-defined secretory epithelium in *B. schunkeana*. The callus anatomy, on the other hand, was similar to *Maxillariella vulcanica* [24] as it was built by a single-layered epidermis, subepidermal layer, and ground parenchyma with collateral vascular bundles. The epidermis on the adaxial (inner) surface stained more strongly for proteins and dihydroxyphenols, but particularly for lipids. The thin, parietal cytoplasm was very dense, stained in the SBB test for lipids presence, with various concentrations of their accumulation in cells. TEM observations showed great content diversity of epidermal cells: different osmiophilic globules, vesicles, and flocculent materials gathered in the cytoplasm, vacuole, and periplasmic space. The combination of histochemical results, TEM observations, and chemical composition allowed us to interpret that the globules present in vacuoles had a phenolic nature, whereas osmiophilic dense cytoplasm was caused by the abundance of lipids. However, Davies & Stpiczyńska [23] noted only inconspicuous lipid droplets in *B. schunkeana*. In *B. picta,* small lipid droplets are present in the cytoplasm and are comparable with the precursors of scent in the osmophores of *Stanhopea* J.Frost *ex* Hook. [23, 29]. In *B. porphyrostele*, parenchyma cells also possessed droplets, but in this case possibly terpenoids, and cuticular pores, similar to the ones that occurred in the osmophores of *Restrepia* Kunth [30] and *Scaphosepalum* Pfitzer [31].

The cytoplasm also contained enlarged nuclei, plastids with lamellae and osmiophilic plastoglobules, numerous rough endoplasmic reticulum, free ribosomes, mitochondria, dictyosomes, and vesicles. Plastoglobules are claimed to be the structures for scent production, and ER localized close to the plastid envelope could transport them to the plasma membrane and through vesicles to the periplasmic space via granulocrine secretion to the exterior [32–34]. The occurrence of periplasmic spaces was already examined in *M. sanguinea* and *M. vulcanica*—narrow ones with vesicles [24], similar, but narrower—in *M. anceps* (*cf.* Figure 6C, [6]), wider—in some *Bulbophyllum* Thouars [35, 36]. This type of route occurs in colleters, nectaries, and cavities for gums, resins, and oils [34]. Secretory materials from the protoplast are gathered in the periplasmic spaces as globules and flocculent material, which is claimed by Paiva [34] that because the cell wall functions as a barrier, secretory materials collect in the periplasmic space. The pressure of accumulated materials interacts on the protoplast. As a result, the cytoplasm is more compressed. The presence of vesicles in the cytoplasm, close to plasmalemma, in periplasmic space, and fully developed dictyosomes provides the next evidence for granulocrine secretion, described in orchids [24, 26, 35, 37]. Furthermore, the enlarged nuclei and numerous mitochondria indicate higher activity in the epidermis. The tiny starch grains sometimes occurred in plastids, but their absence in *B. schunkeana* investigated by Davies & Stpiczyńska [23] could be caused by their utilization during the anthesis. However, in *B. picta*, small starch grains were present [23], same as in *M. sanguinea* and *M. variabilis* [24]. The enlarged nuclei were noticeable in the epidermal layer, which testifies to higher metabolic activity in these cells. In resuts presented by Davies & Stpiczyńska [23] surface secretion was scant and stained weakly with Sudan III, Auramine O, whereas the test with TBO gave a pale blue-grey outcome, which they interpreted as a possible indication of terpenoids. However, our chemical analysis did not reveal the presence of terpenoids. In our view, the TBO test should be treated only as a general histological stain.

In the periplasmic spaces occurred phenolic and lipoid materials. The intravacuolar accumulation of similar osmiophilic precipitates (TEM results) has been previously recorded in *M. variabilis* and *M. vulcanica*, and lipid-rich precipitates (SBB test) in the vacuoles of *M. sanguinea* and *M. variabilis* [24]. The intravacuolar precipitates were reported in *R. notylioglossa* [23]. Intravacuolar osmiophilic bodies and annular profiles are supposed to be involved in the terpenoid synthesis, for example in *M. anceps* [6], or are interpreted as tannin-like materials, as for instance in *Bulbophyllum wendlandianum* (Kraenzl.) Dammer [26], *Epipogium aphyllum* Sw. [38], and *Bulbophyllum weberi* Ames [27].

Davies & Stpiczyńska [23] noted, same as we have, that the volume of secretion produced in *B. schunkeana* was small and stated that it cannot alone account for the glossiness of the labellum. The authors proposed the hypothesis that the thick, outer tangential wall and cuticle, in our examination also observed in the epidermis, in combination, may produce an optical effect, and that pollinators are attracted by the reflective surface. As our results have revealed, the glossy surface of all tepals was caused by the presence of lipids (SBB test) – mainly monoacylglycerols (GC/MS), which are found in the plant cuticular waxes [39]. The waxy substances in the *Maxillaria acuminata* alliance (Maxillariinae) are known to be involved in the process of attracting pollinators, just like the speculum in species of the genus *Ophrys* L. [7] or as in sapromyophilous flowers, where glossy epidermis draws attention to flies imitating wounds or dung [40]. In *M. sanguinea* and *M. vulcanica* the tepals’ brilliance is probably caused by the presence of thick tangential walls and epidermal secretion. The glossiness of the flower is thus a desired feature that influences pollinators’ visits [41].

Test for the presence of catechol-type dihydroxyphenols has indicated them only in plastids, specifically in plastoglobuli [24, 42]. Although the GC/MS analysis revealed the presence of phenol, 3,5-di-tert-butyl-. Phenols are known to be present in the scent of sapromyophilous flowers, for example in the orchid *Bulbophyllum echinolabium* [36] or other plant groups, *e.g. Caralluma europaea* [43]. The osmiophilic secretions on and beneath the cuticle and in plastoglobuli in *Epipactis helleborine* [42] and *B. echinolabium* [36], as well as in *B. schunkeana*, are suggested as phenolic in nature. The fragrance (its phenolic components) is produced in plastoglobuli, then after leaving the plastid, it is transported by means of the ER profiles or individually within the cytoplasm to plasmalemma, and finally to the exterior. Numerous profiles of endoplasmic reticulum adjacent to the plastids with plastoglobuli, vesicles with electron-dense material, and fused with plasmalemma were noted in several other orchid species [26, 44]. In *B. schunkeana*, many rough endoplasmic reticulums were observed in the cytoplasm, close to the plastids and plasmalemma, same as vesicles (sometimes loaded with electron-dense material) in the cytoplasm, close to plasmalemma and in periplasmic space. The hypothesis of the fragrance synthesis and its transport outside the cells yet needs to be proven, however, the involvement of plastids in the synthesis of scent constituents has been already investigated before [24, 35–37, 45, 46].

The electron-dense osmiophilic annular profiles in vacuoles correspond with the phenolic-like inclusions found for instance in the flowers of *Maxillariella variabilis* [24], or leaves of *Anthyllis* L. [47]. Such profiles and flocculent osmiophilic precipitates in vacuoles are also interpreted as phenolic compounds in roots and rhizome of *Echinacea purpurea* (L.) Moench [48]. The osmiophilic contents in vacuoles are the same as those found in the osmophores of *Caesalpinia pulcherrima* (L.) Sw., where phenolic compound began to deposit in the vacuole ([49]; compare: *B. schunkeana*—Figs. 5c, 7c with *C. pulcherrima*—Fig. 4B). The annular osmiophilic profiles are also the initial stage of the fully filled phenolic globules formation (compare Fig. 4c, e with 5e), which were displayed in *C. pulcherrima* (Fig. 4F). The different levels of accumulation of flocculent vacuolar material (Fig. 4a, e) corresponds with the micrographs of the petal osmophores of *Bauhinia rufa* Graham (compare with Fig. 5A-B [49]), where the vacuoles with phenolic compounds were either at the beginning or in an advanced fragmentation stage [49]. Castro & Demarco [50] distinguished two major groups of glands according to their composition. In the first type, the secretory cells are mainly producing phenolics. Contrary to the second group, where phenolics are accompanied by other compounds. In the first type, the phenolic content occurs in fully developed glands and constitutes the main component of secretion of the tissue (epidermis, hypodermis, and also idioblasts, ducts, and sheath around vascular bundles). The latter group consists of cavities, colleters, ducts, laticifers, nuptial nectaries, osmophores, trichomes, and stigma system, in which there is a mixture of phenolics, polysaccharides, terpenes, and other compounds. In floral glands, phenols are associated with pollination, germination of pollen, and elongation of the pollen tube. Moreover, in non-floral organs, phenolic constituents may play a role in chemical defense against ultraviolet rays or pathogens, or as deterrents of herbivores [50].

Phenolic compounds are mainly responsible for the intensive rotten herring scent of flowers. According to the definition provided by Langenheim [51], plant resin is a lipid-soluble mixture, consisting of volatile and nonvolatile compounds, which can be terpenoid (terpenoid resins) and/or phenolic secondary (phenolic resins). In this light, it is almost certain that the secretion in *B. schunkeana* is a phenolic resin. Lipids accumulated in the cells in high amounts can be, however, accountable for the general glossiness The resinous rewards are common in Maxillariinae species [23] and have parallelly evolved in *Maxillariella*, *Mormolyca*, and in the *Heterotaxis/Nitidobulbon/Ornithidium* clade [21]. Nevertheless, they vary in their composition and for example, a resin‐like material of *Heterotaxis* [7, 52] differs from the one of *B. schunkeana* by being rich in mucilage, sugars, amino acids, starch grains, and lipid droplets. Also on the lip surface of *Nitidobulbon* Ojeda, Carnevali & G.A. Romero similar secretion was found [53].

The transverse sections of the lip and other tepals revealed a large number of idioblasts with raphides containing calcium oxalate crystals. They were not found in subepidermis, as generally in orchids [26, 35, 36, 42, 44], but deeper in the tissue (as in *M. vulcanica* [24]). In sepals and lip, they have profusely gathered apically, which could be explained by their biological role. They deter herbivores and prevent tissues from being consumed by them. These observations corroborate with Davies et al. [10] study of a number of *Maxillaria* spp., where in leaf and floral tissues [10] they have reported the presence of raphides and have also suggested that they may be secretory products involved perhaps in discouraging herbivory by invertebrates [10, 54].

### Pollination syndrome—who is the pollinator?

Singer and Cocucci [55] reported that *Brasiliorchis picta* is pollinated by the stingless bee *Trigona spinipes*, however, to our best knowledge, so far the pollination of *B. schunkeana* has never been observed, thus we can type potential pollinators solely indirectly on the basis of the floral morphology and chemical composition*.* Within the compounds detected during GC–MS analysis, several are reported in Pherobase [56] as pheromones, allomones, or attractants (synomones). The following data is sourced from Pherobase [56]. Ethylbenzene is an attractant for some representatives of Diptera (Tephritidae—fruit flies) and Coleoptera (family Scolytidae). It has been described to occur in several plant families, including Orchidaceae (namely *Dendrobium superbum* Rchb. f. and six taxa of *Ophrys* L.). Its odor is described as ethereal, floral, and sweet. p-Xylene is also reported to be present in several plant families and within orchids, in the same *Dendrobium* species and nine *Ophrys* representatives. Its odor is described as cold meat, fat-like. It functions as an attractant for some Diptera (Tephritidae—fruit flies) and Heteroptera (family Miridae). Similar to the previously mentioned, o-Xylene is also an attractant for some representatives of Diptera (fruit flies from the family Tephritidae) and occurs in the same orchid species as p-Xylene. Its odor is described as geranium, oily, fatty, and pungent. Benzoic acid functions as an allomone, attractant, and pheromone for many different animals. It is an attractant for some Diptera (screwworm flies, family Calliphoridae) and Coleoptera (pine shoot beetle, family Curculionidae). Its odor is winey, very weak, and balsamic. Benzoic acid has also been recorded in several plant families and within orchids, it is known to occur in three species of the genus *Phalaenopsis* Blume. Glycerol has the same behavioral functions and it is known to be an attractant for some dipterans (Asian tiger mosquito, family Culicidae). Its odor is characterized as sweet. Hydroxybenzoic acid is a pheromone for some Coleoptera and Hymenoptera (namely Apideae) and allomone for some Coleoptera. Suberic and azelaic acids are pheromones in *Amauris damocles* (Lepidoptera), the latter plays the same role also for *Callosobruchus maculatu*s (Coleoptera). Octadecanoic acid, methyl ester was previously known to occur in Asteraceae Dum. It is an attractant for various species of mites (Astigmata), allomone in some Thysanoptera, Coleoptera, and pheromone in various vertebrates and insects such as Hymenoptera (including *Bombus pomorum*, *B. hypnorum*, *Melipona beecheii*) and some Lepidoptera. Its odor is characterized as oily and waxy. Trehalose is an allomone for some moths (Lepidoptera).

The chemical analysis, therefore, revealed the presence of five semiochemicals that are known to be attractants for some Diptera (mainly families Tephritidae and Calliphoridae; ethylbenzene, p-xylene, o-xylene, benzoic acid, glycerol), which constitutes a strong premise that representatives of this order could be potential pollinators of *B. schunkeana*. It is known that floral scent, color, and texture play an important role in attracting pollinators, such as flies, by for example imitating their brood and feeding sites [57]. According to Davies & Stpiczyńska [23], flowers of both *B. picta* and *B. porphyrostele* are highly fragrant, contrary to *B. schunkeana*. While collecting samples for our research we were able to detect the strong, unpleasant smell, although it seems there is no literature data supporting this observation. However, not all floral fragrances can be detected by every human nose, which may serve as the reason. This strong smell could act as a long-distance attractant as it is known that it helps flies to find the flowers hidden in vegetation [36]. Flowers that are regarded as fly-pollinated are referred to as myophilous or sapromyophilous. The first syndrome is characterized by simple, actinomorphic symmetry, relatively small and bright dull colored (green or yellowish) flowers, with an odor that is slightly sweet or unpleasant for humans [26]. The dark blackish flowers of *B. schunkeana* clearly do not fit this description, contrary to one of the second syndrome in which the flies are attracted by the scents, colors, and surfaces that together imitate flies' natural food sources or their brood sites. The flower colors are primarily dull, greenish to purple-brown, often with spots [26]. Nectar may or may not be produced in sapromyophilous flowers, and in the case of investigated species, it is rather absent although some slight secretory activity has been observed in the column foot. Pollination systems that rely on carrion mimicry are often characterized by the absence of a reward and thereby constitute a form of pollination through deception [58, 59]. It has been, however, demonstrated that flowers that offer meager food rewards are visited more frequently by pollinators than those that lack it completely [41], thus no matter how small quantities of the secreted material are, it is beneficial as food rewards reinforce pollinator foraging behavior. Several floral rewards have evolved independently in the genera that constitute Maxillariinae, for instance, lipoidal rewards evolved in parallel in both *Rudolfiella* Hoehne and *Rhetinantha* M.A, Blanco [21]. Representatives of these genera offer lipoidal rewards, it is waxy in some species of *Rhetinantha* (*e.g.*, *R*. *notylioglossa* (Rchb.f.) M.A. Blanco; [21]. Although we have not confirmed the presence of floral food rewards (including food hairs) in *B. schunkeana*, we have proved the presence of large quantities of lipids in cells, probably the components of wax-like and resinous substances, which are secreted even in the small quantities may constitute a reward itself [6]. Van der Pijl & Dodson [11] suggested that wax and resinous substances may be collected by bees as material for nest building, such as in the already mentioned *Rhetinantha* [21]. As noticed by Davies et al. [7], due to their nutritional value, waxes and resins can also be a source of food substances which would explain why some insects acquire waxy substances from the floral surface.

Vogel [60] and Johnson [61] predicted that flowers pollinated by carrion flies possess traits such as the putrid scent and dull brown coloration. Indeed, in sapromyophilous flowers, the odor is generally strong and disagreeable for humans. It is sometimes compared with the smell of fungi, rotten meat, or further forms of decaying protein [62]. According to Silva et al. [63], fly-pollinated orchids release similar floral scent compositions relating to the compound classes, some of which have been detected in investigated species: n-alkyloketones (in *B. schunkeana* represented by 2,5-di-tert-butyl-1,4-benzoquinone;7,9-di-tert-butyl-1-oxaspiro[4.5]deca-6,9-diene-2,8-dion), n-alkyl-aldehydes, n-alkyl-alcohols, aromatic (in *B. schunkeana*: benzoic acid; hydroxybenzoic acid; ethylbenzene; p-Xylene; o-Xylene; phenol, 3,5-di-tert-butyl) and some terpenes. Odor, the main floral attractant in fly pollination [11, 64, 65], is produced in scent glands (osmophores), that may or may not be morphologically distinguishable from other floral parts [25, 45].

The aforementioned results of the chemical analysis may indicate that *B. schunkeana* could be pollinated by some representatives of the family Tephritidae or Calliphoridae (Diptera). Tephritidae is one of two fly families referred to as fruit flies and it counts nearly 5,000 described species classified within almost 500 genera. The Brazilian state of Espírito Santo, for which *B. schunkeana* is endemic, has one of the largest fruit fly diversities in the country [66]. In general, fruit flies have been extensively studied in the tropics as pests in agricultural areas, but there is not much research conducted in forests with native vegetation [67]. Likewise little is known about the natural history and behavior of fruit flies in nature, therefore underestimating the complexity of fruit fly biology and ecology [68]. The members of the family Calliphoridae are commonly known as blow flies, bluebottles, cluster flies, or greenbottles. Their distribution is cosmopolitan and over 1,000 species and about 150 genera have been recognized so far [69, 70]. Adult blow flies are known to be effective pollinators [71], and since they are generally necrophagous and use decomposing organic matter for their proliferation, they are usually attracted to flowers with strong odors resembling *i.e.* rotting meat. The calliphorids have a wide variety of habits and can be found visiting flowers, excrement, termite nest mounds, and driver-ant columns, as well as in decomposing plants and animals ([72] and references therein). The diversity of the family, biology, and ecology of its representatives in tropical biomes seems to be a major holdback to the knowledge of this group in Latin America and their role as pollinators. The first record of Calliphoridae as pollinators of an orchid species (*Epidendrum tridactylum* Lindl.) in the Americas has been reported only nine years ago by Pansarin & Pansarin [73]. The determination of whether representatives of either of these groups (or perhaps both?) are pollinators of *B. schunkeana* solely on the basis of the obtained results would be serious malfeasance. Van der Niet et al. [74] however rightly noted that it is necessary to reinterpret the sapromyiophilous pollination syndrome from one considered to be generalized for ‘carrion flies’ to one that may reflect several specialized interactions involving different groups of flies. In their study, the authors have underlined the need to define specialization in terms of the proportion of insect species in local assemblages that could potentially pollinate flowers, and in this view—based on chemical and anatomical results, it is probable that both fruit and blow flies could serve as pollinators.

Our findings seem to support this hypothesis, however, there is another theory that should be spelled out and that points to vulture bees, also known as carrion bees, as possible pollinators. This specific group of stingless bees (Meliponini) counts three *Trigona* species that have been referred to as obligate necrophages that use flesh as a protein source instead of pollen [75, 76], these are *T. crassipes*, *T. necrophaga,* and *T. hypogea*. In Brazil, only *T. crassipes* and *T. hypogea* occur naturally. So far we were not able to find any records of orchid pollination by these insects, nor the dedicated pollination syndrome has been segregated for carrion bees. It seems safe to assume that it would greatly resemble sapromyophyly rather than melittophily (bee pollination). Some studies of the relation between orchids and bees have proved that orchids attract and deceive social bees with aggregation pheromone mimics, as they contain the same components that are products of the bees’ glands [77, 78]. As stated by Roubik [5] while mimicry of odor trails or pheromones attracting foraging nestmates may appear in the fragrances of orchids attractive to Meliponini visitors, this problem may be more complex. He suggested that meliponine interest in orchid flowers may be caused by odors mimicking bee exocrine gland chemicals, including pheromones used in foraging or nest defense. Meliponine pheromones are released mainly from mandibular glands [79, 80] and often comprise a mixture of 2-alcohols, 2-ketones, and esters [81]. In our analysis, we did not find semiochemicals that are bee attractants, but it indeed revealed the presence of three compounds that are known to be pheromones for some Hymenoptera: p-Xylene, hydroxybenzoic acid (particularly for Apideae), octadecanoic acid, methyl ester (namely for some *Bombus* species and *Melipona beecheii*). According to Francke et al. [82] semiochemicals present in the cephalic secretions of *T. hypogea* workers are heptan-2-ol (pheromone), 8OH, octan-2-ol, nonan-2-ol, caprylic acid, pelargonic acid, oleic acid, stearic acid, octyl octanoate. None of these has been, however, detected in our study. *T. hypogea* has two distinct food sources: carrion (protein), and fruits and extrafloral nectaries (sugars) [76]. Similar to other Meliponinae, there are distinct kinds of storage pots for honey and for protein [76]. The food specialization of *T. hypoge* does not seem to be accompanied by any significant changes in morphology or behavior, thus it is not impossible that it may serve as a pollinator. However, according to Camargo (cited in [76]) a year-round sampling of flowers in an area where native nests of *T. hypogea* were present confirmed that such bees never visit flowers. The bees have been, nevertheless, spotted foraging on medium-ripe fruits of *Eugenia jambolana* Lam. which contain tannins and are either added to the resin deposits or directly applied to nest crevices [76]. Tannins are known for their antibacterial properties, and as Noll et al. [76] points out, they may play an important role in connection with the necrophagous feeding habits of *T. hypogea*, which exposes the bees to several kinds of microbial contaminants. Assuming that vulture bees could indeed be the pollinators, arises the question, of whether this could be somehow connected to the high concentration of 2,5-di-tert-butyl-1,4-benzoquinone detected in floral extracts of *B. schunkeana* and its antibacterial properties (further described below). Since *Tigona* nests are constructed from wax they produce and plant resins they collect, maybe they indeed visit flowers of *B. schunkeana* to collect antimicrobial resins.

Unfortunately, the question of which scenario (pollination by flies vs. vulture bees) is the correct one, or if there is yet another player, will remain open until extensive in situ field studies will be conducted.

### Biological activity

It is not a secret that orchids are widely used in traditional medicine for the treatment of different health conditions such as for instance hypertension, tuberculosis, paralysis, stomach disorders, or arthritis [83]. Some of them are used as emetics, aphrodisiacs, vermifuges, bronchodilators, or sex stimulators, or to treat scorpion stings and snake bites [84]. Representatives of *Maxillaria *sensu lato are not an exception and are also widely used in traditional medicine for their antispasmodic and anti-inflammatory activities [85, 86]. Waratchareeyakul et al. [83] have for example reported vasorelaxant activity of stilbenoid and phenanthrene derivatives from *Brasiliorchis porphyrostele* (Rchb.f). The major compounds identified in dichloromethane extract from *B. schunkeana* were 7,9-di-tert-butyl-1-oxaspiro(4,5)deca-6,9-diene-2,8-dione (77.06%) and 2,5-di-tert-butyl-1,4-benzoquinone (16.65%). The first one is referred to as a degradation product of primary phenolic antioxidants [87, 88] and has been found naturally occurring in small quantities (usually < 3%) in aerial parts of *Gmelina asiatica* Linn (Verbenaceae; [89]), in the extract of tree fern *Cyathea nilgirensis* Holttum [90], rhizomes of *Cyperus rotundus* L. [91], *Cuscuta reflexa* Roxb. [92], *Cordia sebestena* L. [93], fruit skin of *Mangifera indica* L. [94], and three orchid species *Bulbophyllum echinolabium* J.J. Sm. [36], *Maxillariella sanguinea* (Rolfe) M.A. Blanco & Carnevali [24], *M. vulcanica* (F. Lehm. & Kraenzl.) M.A. Blanco & Carnevali [24], and *M. tenuifolia* (Lindl.) M.A. Blanco & Carnevali [95]. In the latest one, its concentration exceeded 10%. Rukhsana et al. [96] reported that 7,9-Di-tert-butyl-1-oxaspiro(4,5)deca-6,9-diene-2,8-dione has steroidal antimineralocorticoid activity and anti-androgen, weak progesterone properties, some indirect estrogen, and glucocorticoid effect. According to Rao et al. [97], it is used primarily as a diuretic and antihypertensive, to treat heart failure, ascites in patients with liver disease, lowering hypertension, hypokalemia, secondary hyperaldosteronism (such as occurs with hepatic cirrhosis), and Conn's syndrome (primary hyperaldosteronism). What is more, it is frequently used to treat a variety of skin conditions including hirsutism, androgenic alopecia, acne, and seborrhea in females and male pattern baldness. The second of the major compounds, 2,5-di-tert-butyl-1,4-benzoquinone (DTBBQ) is a member of p-quinones and a member of benzoquinones. According to Gopal et al. [98] it is a biologically active quinone-based pigment. It has been originally isolated from marine *Streptomyces* sp. VITVSK1 and was also detected in low amounts in *Bacillus* spp (0.512%; [99]) as well as orchids *B. echinolabium* (2.65%; [36]) and *Maxillariella vulcanica* (F. Lehm. & Kraenzl.) M.A. Blanco & Carnevali (0.07%; [24]). DTBBQ is known as a potent antibacterial agent which inhibits the RNA polymerase enzyme [100, 101]. According to Johnson-Ajinwo et al. [102], it also shows potent antiplasmodial activity. It appears that the quantity of both compounds detected in *B. schunkeana* is unprecedentedly high compared to other sources and further investigation should take place to establish if this unique orchid could be in the future more effective source of these two chemicals.

## Materials and methods

Fresh flowers at the different stages of anthesis were collected from the plants cultivated in the greenhouses of the Faculty of Biology, University of Gdańsk, Poland (voucher number ML231407, ML182003), and in the facilities of Currlin Orchideen, Uffenheim, Germany (voucher number CUR202109). Species identification has been performed by Monika M. Lipińska and Dariusz L. Szlachetko with the methods of the classical taxonomy. For the SEM analysis we used four flowers (vouchers: ML231407, ML182003, DLS *s.n.*, CUR202109), for TEM we used three flowers (vouchers: ML231407, ML182003, CUR202109), histochemistry two flowers (vouchers: ML231407, CUR202109), and chemical analysis three flowers (vouchers: ML231407, ML182003, CUR202109). Voucher specimens have been deposited in the UGDA Herbarium. Research complied with relevant institutional, national, and international guidelines and legislation.

For the scanning electron microscopy (SEM) samples were preserved in a standard mixture of chemical reagents: glutaraldehyde (GA, 2,5% (v/v)) in 0,05 M cacodylate buffer (pH 7,0), then dehydrated in ethanol (from 10 to 100%), dried in critical point using liquid CO_2_, and before observation in a scanning electron microscope (Philips XL-30) covered by a film of gold.

For histochemistry, the flowers were treated in a fixative mixture: glutaraldehyde (GA, 2.5% (v/v)) in 0.05 M cacodylate buffer (pH = 7.0). Then the floral parts were washed out with a cacodylate buffer and dehydrated in the ethanol (from 10 to 100%). The methylmethacrylate-based resin (Technovit 7100, Heraeus Kulzer GmbH) was used to embed the material at the final step before cutting. 5–7 μm thick sections were cut using a microtome (Leica EM UC 7) and mounted on glass slides. For control, the sections were stained with aqueous Toluidine Blue O (0.05% (w/v), TBO, C.I. 52,040) [103, 104]. This metachromatic stain gives different levels of blue color for cell organelles, according to their composition, and this is the reason for using it as a general histological stain [23]. The test of Aniline Blue Black (ABB, C.I. 20,470) and the Periodic Acid-Schiff reaction (PAS) were used for the presence of water-insoluble proteins and polysaccharides, respectively [105]. The test with a 0.05% (w/v) aqueous Ruthenium Red (C.I. 77,800) solution revealed the pectic acids/mucilage [106]. A 10% (w/v) aqueous solution of FeCl_3_ indicated catechol-type dihydroxyphenols [107]. The floral sections were studied and photographed with a Nikon Eclipse E 800 light microscope equipped with a Nikon DS-5 Mc camera using Lucia Image software (University of Gdańsk, Poland) and differential interference contrast (DIC). For detection of the cuticle peculiarly unsaturated cutin precursors and acidic waxes [107], 0.01% (w/v) solution of Auramine O (C.I. 41,000) in 0.05 M buffer Tris/HCl (pH = 7.2) was applied. The observations were done with a Nikon Eclipse E800 fluorescence microscope, equipped with filter B-2A (EX 450–490 nm, DM 505 nm, BA 520 nm).

For examination in transmission electron microscopy (TEM), after the fixation in 2,5% (v/v), glutaraldehyde (GA) in 0,05 M cacodylate buffer (pH 7,0), the floral tissue was post-fixed overnight in 1% OsO_4_ in the cacodylate buffer. Then, the dehydration of the labellar fragments was allowed by means of graded acetone series and finally embedded in Spurr’s resin. Ultrathin slides (60 nm) were prepared with an ultramicrotome (Leica UC7) and studied in an FEI Tecnai Spirit BioTWIN transmission electron microscope at 120 kV.

For chemical analyses, the liquid was carefully collected from flowers using several small pads of glass wool and then extracted in 10 ml methanol. Whole flowers were subjected to sequential organic solvent extraction. First, non-polar compounds were isolated in 10 ml dichloromethane for 20 s, then carbohydrates were extracted by dipping flowers for 30 s in 10 ml methanol. Extracts were then stored at 4 °C prior to analysis.

The dichloromethane extract was evaporated at room temperature to ca. 0.3 ml under a stream of nitrogen. An aliquot of methanol extract was evaporated to dryness under a stream of nitrogen. The methanol extract was subjected to a derivatization process. The sample was silylated with 100 ul of a mixture of 99% N,O-Bis(trimethylsilyl) trifluoroacetamide (BSTFA) and 1% Trimethylchlorosilane (TMCS) for 1 h at 100 °C. Silylated and native samples (the dichloromethane extract) were analyzed using gas chromatography-mass spectrometry (GC–MS). Gas chromatography–mass spectrometry measurements were carried out by a Shimadzu QP-2010SE system (Shimadzu, Kyoto, Japan). The samples were introduced through the gas chromatograph equipped with a 30 × 0.25 mm i.d., film thickness 0.25 μm, ZB-5 capillary column (Phenomenex, USA). For silylated samples, the oven temperature of 80 °C (held for 10 min) was increased to 310 °C at 4 °C/min. For dichloromethane extract, the column temperature was programmed from 40 °C (isothermal for 3 min) to 310 °C at 4 °C/min. The injector temperature was 310 °C and the carrier gas was helium. The ion source was maintained at 210 °C. The split ratio was 1:20. The injection volume was 1 μL. The GC–MS analysis was performed in triplicate for each sample.

## Supplementary Information

**Additional file 1:** **Fig. S1.** Results of histochemical tests performed on the end of the callus (1/3 of the lip length): **a**-**b** epidermis and some parenchyma cells slightly stained for proteins (ABB). **c** few and tiny starch grains in the epidermis (PAS). The idioblasts with raphides are indicated by arrows through transverse sections of **d** lip base with flat callus to the beginning of the raising callus. **e** callus (from the middle part to the abaxial surface). **f** lip apex. **g** dihydroxyphenols (FeCl_3_ test) stained only in plastids, possibly in plastoglobules. **h** no mucilage/pectic acids on the surface (Ruthenium Red). *ab* - abaxial (outer) surface, *ad* - adaxial (inner) surface, *n* - nucleus, *pa* - parenchyma, *r* - idioblasts with raphides, *vb* - vascular bundle.

**Additional file 2:** **Fig. S2.** The further observations of the epidermis of the lip base with flat callus from transmission electron microscopy (TEM) showing: **a** narrow or expanded periplasmic spaces with varying quantities and sizes of globules and vesicles, thick outer tangential cell wall, stretched cuticle caused by accumulated underneath secretory products and some of them visible on its surface (*arrows*). **b** magnification of **a**, periplasmic space with secretory material and vesicles, dense cytoplasm with organelles: here visible profiles of RER, free ribosomes, mitochondria. **c** different cells of the epidermis with stretched cuticle (*arrow*), periplasmic space, and osmiophilic annular profiles in the vacuole (*asterisks*). **d** magnification of c, periplasmic space with vesicles, dense cytoplasm with abundant RER, osmiophilic material in the vacuole. **e** magnification of **c**, vacuolar fragmentation, and osmiophilic annular profiles (*asterisk*), sometimes disintegrated (*arrows*), in the cytoplasm: plastid with lamellae and starch grain. **f** magnification of **e**, in cytoplasm mitochondria, RER, plastid with lamellae and plastoglobules. *cw* - cell wall, *m* - mitochondrion, *p* - plastid, *ph* - phenolic content, *ps* - periplasmic space, *RER* - rough endoplasmic reticulum, *st* - starch grains, *va* - vacuole, *ve* - vesicle.

**Additional file 3: Fig. S3.** The TEM results presenting callus epidermis (1/3 of the lip length): **a** magnification of **6f**. **b** large nucleus surrounded by numerous plastids and vesicles, osmiophilic phenolic content in the vacuole (*arrows*). Lip apex: **c** the heterogenous remnants of secretion with osmiophilic phenolic content (also gathered in the vacuole, *white arrows*) in the papillae. **d** magnification of **c**. **e** the osmiophilic phenolic globules and phenolic content in vacuoles and vacuolar fragmentation.** f** magnification of **e**. *cw* - cell wall, *d* - dictyosome, *n* - nucleus, *p* - plastid, *ph* - phenolic content, *ps* - periplasmic space, *st* - starch grain, *va* - vacuole, *ve* - vesicle.

**Additional file 4: Fig. S4.** Histochemical tests of column foot: **a** the single-layered epidermis, parenchyma with collateral vascular bundles. **b** test for the presence of proteins (ABB). **c** few and tiny starch grains (PAS). **d** dihydroxyphenols in the epidermis (FeCl_3_). *ab* - abaxial (outer) surface, *ad* - adaxial (inner) surface, *n* - nucleus, *vb* - vascular bundle.

## Data Availability

All additional images supporting the presented results are included as supplementary files.
