# Supplementary material for: Floral attractants in the black orchid Brasiliorchis schunkeana (Orchidaceae, Maxillariinae): clues for presumed sapromyophily and potential antimicrobial activity
Source: BMC Plant Biol. 2022 Dec 10;22:575. doi: 10.1186/s12870-022-03944-8 (PMC9737770; doi:10.1186/s12870-022-03944-8)
Supplement: Supplementary file 1 — Additional file 1: Fig. S1. Results of histochemical tests performed on the end of the callus (1/3 of the lip length): a-b epidermis and some parenchyma cells slightly stained for proteins (ABB). c few and tiny starch grains in the epidermis (PAS). The idioblasts with raphides are indicated by arrows through transverse sections of d lip base with flat callus to the beginning of the raising callus. e callus (from the middle part to the abaxial surface). f lip apex. g dihydroxyphenols (FeCl3 test) stained only in plastids, possibly in plastoglobules. h no mucilage/pectic acids on the surface (Ruthenium Red). ab - abaxial (outer) surface, ad - adaxial (inner) surface, n - nucleus, pa - parenchyma, r - idioblasts with raphides, vb - vascular bundle. [file 12870_2022_3944_MOESM1_ESM.pdf]

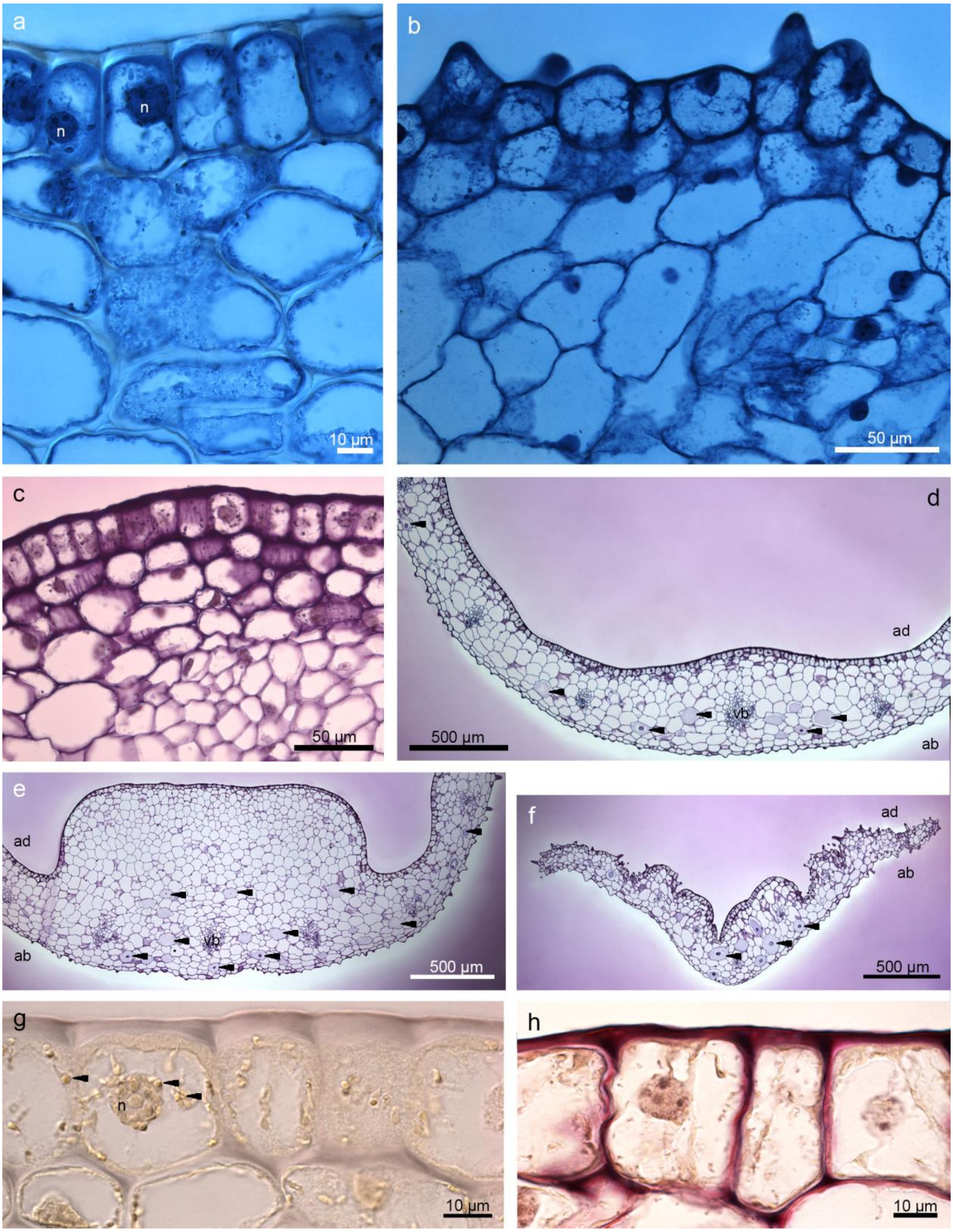

Fig. S1. Results of histochemical tests performed on the end of the callus (⅓ of the lip length): **a-b** epidermis and some parenchyma cells slightly stained for proteins (ABB). **c** few and tiny starch grains in the epidermis (PAS). The idioblasts with raphides are indicated by *arrows* through transverse sections of **d** lip base with flat callus to the beginning of the raising callus. **e** callus (from the middle part to the abaxial surface). **f** lip apex. **g** dihydroxyphenols ($FeCl_3$ test) stained only in plastids, possibly in plastoglobules. **h** no mucilage/pectic acids on the

surface (Ruthenium Red). *ab* - abaxial (outer) surface, *ad* - adaxial (inner) surface, *n* - nucleus, *pa* - parenchyma, *r* - idioblasts with raphides, *vb* - vascular bundle.
