# Supplementary material for: Floral attractants in the black orchid Brasiliorchis schunkeana (Orchidaceae, Maxillariinae): clues for presumed sapromyophily and potential antimicrobial activity
Source: BMC Plant Biol. 2022 Dec 10;22:575. doi: 10.1186/s12870-022-03944-8 (PMC9737770; doi:10.1186/s12870-022-03944-8)
Supplement: Supplementary file 2 — Additional file 2: Fig. S2. The further observations of the epidermis of the lip base with flat callus from transmission electron microscopy (TEM) showing: a narrow or expanded periplasmic spaces with varying quantities and sizes of globules and vesicles, thick outer tangential cell wall, stretched cuticle caused by accumulated underneath secretory products and some of them visible on its surface (arrows). b magnification of a, periplasmic space with secretory material and vesicles, dense cytoplasm with organelles: here visible profiles of RER, free ribosomes, mitochondria. c different cells of the epidermis with stretched cuticle (arrow), periplasmic space, and osmiophilic annular profiles in the vacuole (asterisks). d magnification of c, periplasmic space with vesicles, dense cytoplasm with abundant RER, osmiophilic material in the vacuole. e magnification of c, vacuolar fragmentation, and osmiophilic annular profiles (asterisk), sometimes disintegrated (arrows), in the cytoplasm: plastid with lamellae and starch grain. f magnification of e, in cytoplasm mitochondria, RER, plastid with lamellae and plastoglobules. cw - cell wall, m - mitochondrion, p - plastid, ph - phenolic content, ps - periplasmic space, RER - rough endoplasmic reticulum, st - starch grains, va - vacuole, ve - vesicle. [file 12870_2022_3944_MOESM2_ESM.pdf]

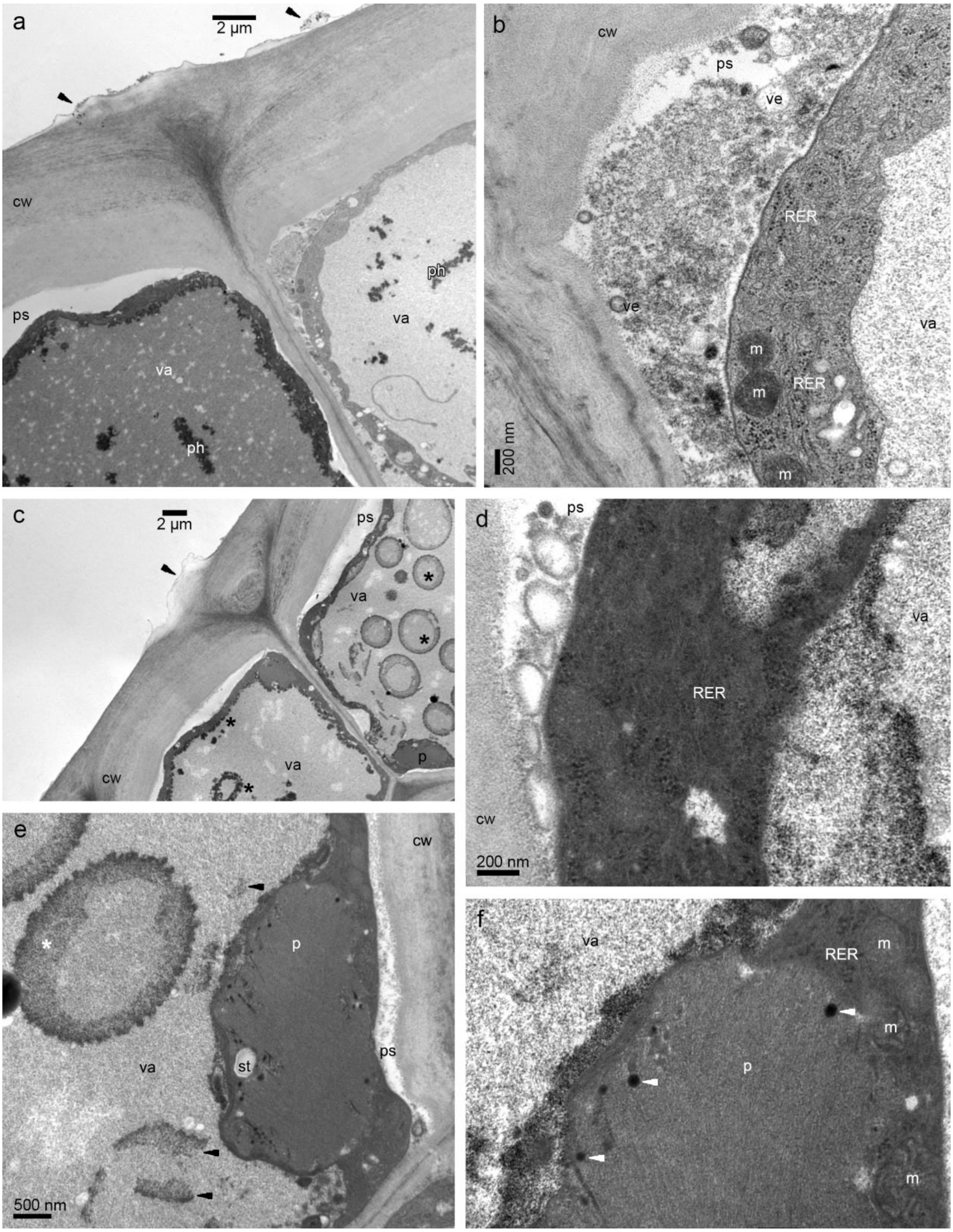

Fig. S2. The further observations of the epidermis of the lip base with flat callus from transmission electron microscopy (TEM) showing: **a** narrow or expanded periplasmic spaces with varying quantities and sizes of globules and vesicles, thick outer tangential cell wall, stretched cuticle caused by accumulated underneath secretory products and some of them

visible on its surface (*arrows*). **b** magnification of **a**, periplasmic space with secretory material and vesicles, dense cytoplasm with organelles: here visible profiles of RER, free ribosomes, mitochondria. **c** different cells of the epidermis with stretched cuticle (*arrow*), periplasmic space, and osmiophilic annular profiles in the vacuole (*asterisks*). **d** magnification of **c**, periplasmic space with vesicles, dense cytoplasm with abundant RER, osmiophilic material in the vacuole. **e** magnification of **c**, vacuolar fragmentation, and osmiophilic annular profiles (*asterisk*), sometimes disintegrated (*arrows*), in the cytoplasm: plastid with lamellae and starch grain. **f** magnification of **e**, in cytoplasm mitochondria, RER, plastid with lamellae and plastoglobules. *cw* - cell wall, *m* - mitochondrion, *p* - plastid, *ph* - phenolic content, *ps* - periplasmic space, *RER* - rough endoplasmic reticulum, *st* - starch grains, *va* - vacuole, *ve* - vesicle.
