# Supplementary material for: Floral attractants in the black orchid Brasiliorchis schunkeana (Orchidaceae, Maxillariinae): clues for presumed sapromyophily and potential antimicrobial activity
Source: BMC Plant Biol. 2022 Dec 10;22:575. doi: 10.1186/s12870-022-03944-8 (PMC9737770; doi:10.1186/s12870-022-03944-8)
Supplement: Supplementary file 3 — Additional file 3: Fig. S3. The TEM results presenting callus epidermis (1/3 of the lip length): a magnification of 6f. b large nucleus surrounded by numerous plastids and vesicles, osmiophilic phenolic content in the vacuole (arrows). Lip apex: c the heterogenous remnants of secretion with osmiophilic phenolic content (also gathered in the vacuole, white arrows) in the papillae. d magnification of c. e the osmiophilic phenolic globules and phenolic content in vacuoles and vacuolar fragmentation. f magnification of e. cw - cell wall, d - dictyosome, n - nucleus, p - plastid, ph - phenolic content, ps - periplasmic space, st - starch grain, va - vacuole, ve - vesicle. [file 12870_2022_3944_MOESM3_ESM.pdf]

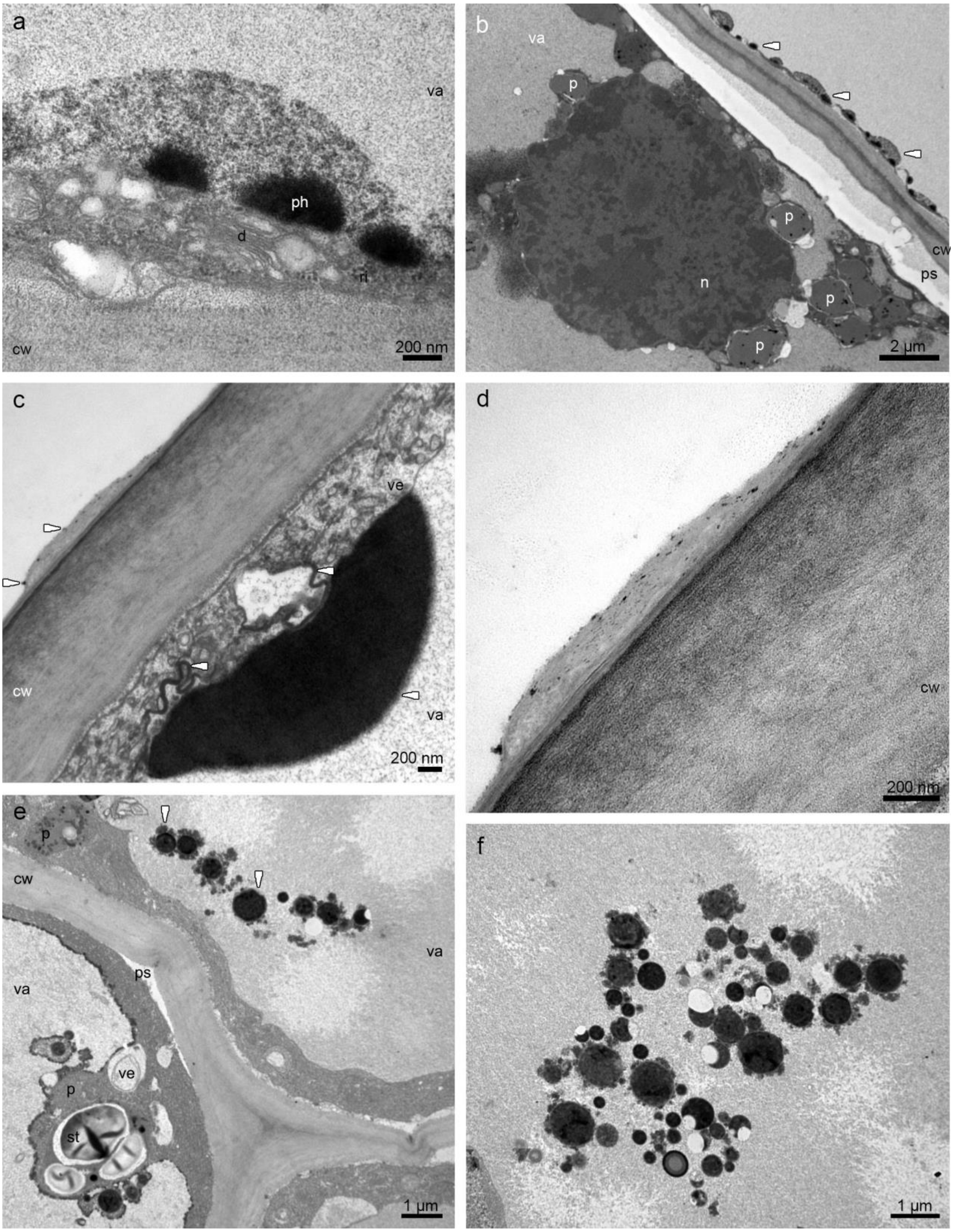

Fig. S3. The TEM results presenting callus epidermis (⅓ of the lip length): **a** magnification of **6f**. **b** large nucleus surrounded by numerous plastids and vesicles, osmiophilic phenolic content in the vacuole (*arrows*). Lip apex: **c** the heterogenous remnants of secretion with osmiophilic phenolic content (also gathered in the vacuole, *white arrows*) in the papillae. **d**

magnification of **c**. **e** the osmiophilic phenolic globules and phenolic content in vacuoles and vacuolar fragmentation. **f** magnification of **e**. *cw* - cell wall, *d* - dictyosome, *n* - nucleus, *p* - plastid, *ph* - phenolic content, *ps* - periplasmic space, *st* - starch grain, *va* - vacuole, *ve* - vesicle.
