# Supplementary material for: Floral attractants in the black orchid Brasiliorchis schunkeana (Orchidaceae, Maxillariinae): clues for presumed sapromyophily and potential antimicrobial activity
Source: BMC Plant Biol. 2022 Dec 10;22:575. doi: 10.1186/s12870-022-03944-8 (PMC9737770; doi:10.1186/s12870-022-03944-8)
Supplement: Supplementary file 4 — Additional file 4: Fig. S4. Histochemical tests of column foot: a the single-layered epidermis, parenchyma with collateral vascular bundles. b test for the presence of proteins (ABB). c few and tiny starch grains (PAS). d dihydroxyphenols in the epidermis (FeCl3). ab - abaxial (outer) surface, ad - adaxial (inner) surface, n - nucleus, vb - vascular bundle. [file 12870_2022_3944_MOESM4_ESM.pdf]

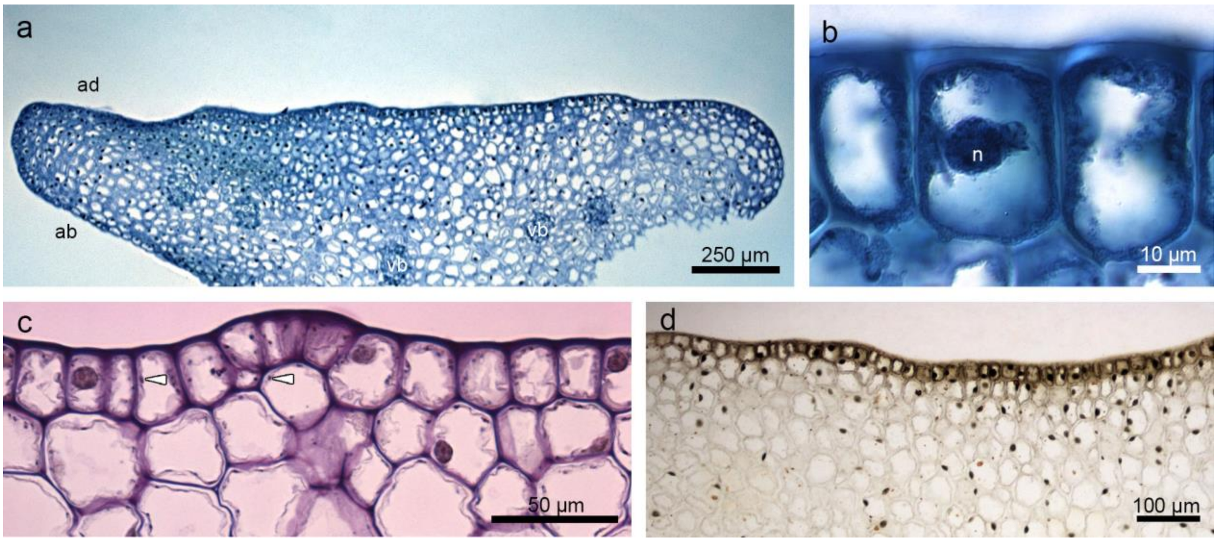

Fig. S4. Histochemical tests of column foot: **a** the single-layered epidermis, parenchyma with collateral vascular bundles. **b** test for the presence of proteins (ABB). **c** few and tiny starch grains (PAS). **d** dihydroxyphenols in the epidermis ($FeCl_3$). *ab* - abaxial (outer) surface, *ad* - adaxial (inner) surface, *n* - nucleus, *vb* - vascular bundle.
